# Modeling the persistence of *Opisthorchis viverrini* worm burden after mass-drug administration and education campaigns with systematic adherence

**DOI:** 10.1371/journal.pntd.0011362

**Published:** 2024-02-29

**Authors:** Lars Kamber, Christine Bürli, Helmut Harbrecht, Peter Odermatt, Somphou Sayasone, Nakul Chitnis

**Affiliations:** 1 Swiss Tropical and Public Health Institute, Allschwil, Switzerland; 2 University of Basel, Basel, Switzerland; 3 Department of Mathematics and Computer Science, University of Basel, Basel, Switzerland; 4 Lao Tropical and Public Health Institute, Vientiane, Lao People’s Democratic Republic; University of Liverpool, UNITED KINGDOM

## Abstract

*Opisthorchis viverrini* is a parasitic liver fluke contracted by consumption of raw fish, which affects over 10 million people in Southeast Asia despite sustained control efforts. Chronic infections are a risk factor for the often fatal bile duct cancer, cholangiocarcinoma. Previous modeling predicted rapid elimination of *O. viverrini* following yearly mass drug administration (MDA) campaigns. However, field data collected in affected populations shows persistence of infection, including heavy worm burden, after many years of repeated interventions. A plausible explanation for this observation is systematic adherence of individuals in health campaigns, such as MDA and education, with some individuals consistently missing treatment.

We developed an agent-based model of *O. viverrini* which allows us to introduce various heterogeneities including systematic adherence to MDA and education campaigns at the individual level. We validate the agent-based model by comparing it to a previously published population-based model. We estimate the degree of systematic adherence to MDA and education campaigns indirectly, using epidemiological data collected in Lao PDR before and after 5 years of repeated MDA, education and sanitation improvement campaigns. We predict the impact of interventions deployed singly and in combination, with and without the estimated systematic adherence.

We show how systematic adherence can substantially increase the time required to achieve reductions in worm burden. However, we predict that yearly MDA campaigns alone can result in a strong reduction of moderate and heavy worm burden, even under systematic adherence. We predict latrines and education campaigns to be particularly important for the reduction in overall prevalence, and therefore, ultimately, elimination. Our findings show how systematic adherence can explain the observed persistence of worm burden; while emphasizing the benefit of interventions for the entire population, even under systematic adherence. At the same time, the results highlight the substantial opportunity to further reduce worm burden if patterns of systematic adherence can be overcome.

## Author summary

*Opisthorchis viverrini* is a liver parasite contracted by consumption of raw fish which affects over 10 million people in Southeast Asia despite sustained control efforts, with heavy infections being a major risk factor for often fatal bile duct cancer. Efforts to reduce the worm burden in populations include education of individuals on the safe consumption of fish meals, improvement of sanitation to interrupt the parasitic life cycle and mass drug administration campaigns, where ideally all targeted individuals of a population receive a drug against the parasite without a prior test of whether they are infected.

We present a new agent-based mathematical model of *Opisthorchis viverrini* transmission and control. This model allows us to include systematic adherence to interventions, which describes the pattern of some individuals consistently being missed by interventions aimed at an entire (sub-)population. Our findings show how systematic adherence can explain the observed persistence of worm burden, but how interventions can be greatly beneficial even for individuals that systematically miss treatment. At the same time, we show the substantial opportunity to further reduce worm burden if patterns of systematic adherence can be overcome.

## 1 Introduction

*Opisthorchis viverrini* is a parasitic liver fluke endemic to the Greater Mekong Subregion (GMS) in Southeast Asia [1]. While substantial reductions in *O. viverrini* prevalence were achieved over recent decades, a total of 12.4 million individuals in the GMS were estimated to still be infected in 2018 [2–4]. Infections with *O. viverrini* can be asymptomatic [5] but frequently cause liver morbidity and, especially after sustained heavy infection, cholangiocarcinoma, a malignancy with poor prognosis [6–11].

Humans and other mammals, such as dogs and cats, serve as definitive hosts of *O. viverrini* [12, 13]. The mature parasite resides in the bile duct producing eggs which reach the environment with the hosts’ stool. The eggs are ingested by freshwater snails of the genus *Bithynia*. The parasite hatches inside the snails and develops into *cercariae*. The *cercariae* shed from the snails, penetrate the skin of fish from the carp family and develop into *metacercariae* inside the fish. When definitive hosts consume infected fish in raw or undercooked form, the *metacercariae* migrate to the hosts’ bile duct and develop into adult worms that can live for many years. The intensity of liver fluke infection is highly heterogeneous in populations where *O. viverrini* is endemic, with relatively few individuals exhibiting very heavy worm burden [14]. This pattern, also referred to as *aggregation*, is typical for macroparasitic infections and has been explained by differences in exposure, susceptibility and immune response of individuals [15, 16].

*O. viverrini* infection can effectively be treated with praziquantel when dosed correctly [17]. Non-pharmaceutical interventions include education campaigns on safe fish consumption and use of sanitation, improvement of sanitation, snail control and safe fish farming. Mass drug administration (MDA) campaigns with praziquantel are conducted regularly along with other control programs in endemic areas [18], but infection and subsequent morbidity prevails despite these efforts [1, 14, 19]. Possible causes are incomplete MDA coverage [18] and continued consumption of raw and undercooked fish leading to substantial reinfection among treated individuals [20–23].

Usually, not all eligible individuals in a population targeted by an MDA campaign take the drug in the end. The terminology used in the literature to describe this phenomenon is not unique [24]. Here, we use the term *coverage* to describe the proportion of eligible individuals that take the drug during an MDA campaign. If not all individuals have the same probability of taking the drug, and some are more likely to take it than others over multiple MDA campaigns, we refer to this as *systematic adherence* and say that individuals *adhere* to the campaign if they do take the drug.

Measuring systematic adherence empirically is difficult as it ideally involves repeated direct observation of individuals taking treatment over multiple MDA campaigns and hence evidence on the extent of systematic adherence is limited [24]. Empirical estimates mostly based on surveys have estimated systematic adherence in terms of adjusted odds ratio of (non)-adherence based on previous (non)-adherence with values roughly ranging between 1.5 and 10 [25–28]. We also expect the extent of systematic adherence to be highly setting- and treatment-specific. Multiple ways of including systematic adherence to MDA in infectious disease models have been proposed and implemented [29, 30]. These include partitioning the population into a part that always adheres and a part which never adheres, assigning probabilities of adherence to individuals that stay fixed over multiple MDA campaign or correlation schemes which result in individual probabilities of adherence based on past adherence [29].

In previous work, we developed various population-based models (PBMs) for *O. viverrini* transmission and control. These are ordinary and partial differential equation models that track the mean worm burden in humans and reservoir hosts, and the prevalence of infection in intermediate hosts. The main conclusions from PBM studies are that elimination can be achieved by targeting humans only (as opposed to reservoir hosts such as cats and dogs) with repeated interventions within a few years [31, 32], but that covering all age groups is necessary to achieve this goal [33]. Other modeling work on *O. viverrini* has focused on the effect of ecological and climate factors on transmission [34, 35]. Furthermore, several compartmental models for the transmission and control of *Clonorchis sinensis*, a parasite with the same life cycle as *O. viverrini*, have been published [36–40].

While PBMs can provide distributions of worm burden as an output based on mean worm burden [15], they usually assume homogeneity of individuals when it comes to susceptibility to infection, behavior, and adherence to interventions. PBMs can accommodate for heterogeneity to some degree by adding compartments for each combination of considered characteristics. For example, separate compartments were added for individuals with different propensity to consume undercooked fish in a PBM for *C. sinensis* [38]. However, when modeling multiple sources of heterogeneity simultaneously, the number of required compartments with this approach grows exponentially.

We developed an agent-based model (ABM) of *O. viverrini* which allows us to explicitly add heterogeneities, including systematic adherence. We fit the ABM in equilibrium to data collected in 2012 in two villages in southern Lao PDR. We compare the equilibrium state and intervention impact predictions of the ABM for a combination of MDA, education, and sanitation improvement without systematic adherence to the output of a previously published PBM. We then introduce systematic adherence to MDA and education campaigns in the ABM. Using data collected in 2018 in the same villages after MDA, education, and sanitation improvement were implemented, we fit the extent of systematic adherence in the model. We estimate the contribution of these interventions with the fitted systematic adherence on changes in mean worm burden and distribution of worm burden. We finally predict the impact of deploying MDA, education and latrines in combination and isolation under non-systematic and systematic adherence to assess their potential for reduction of worm burden in the future.

## 2 Methods

### 2.1 Data

**Fig 1:**
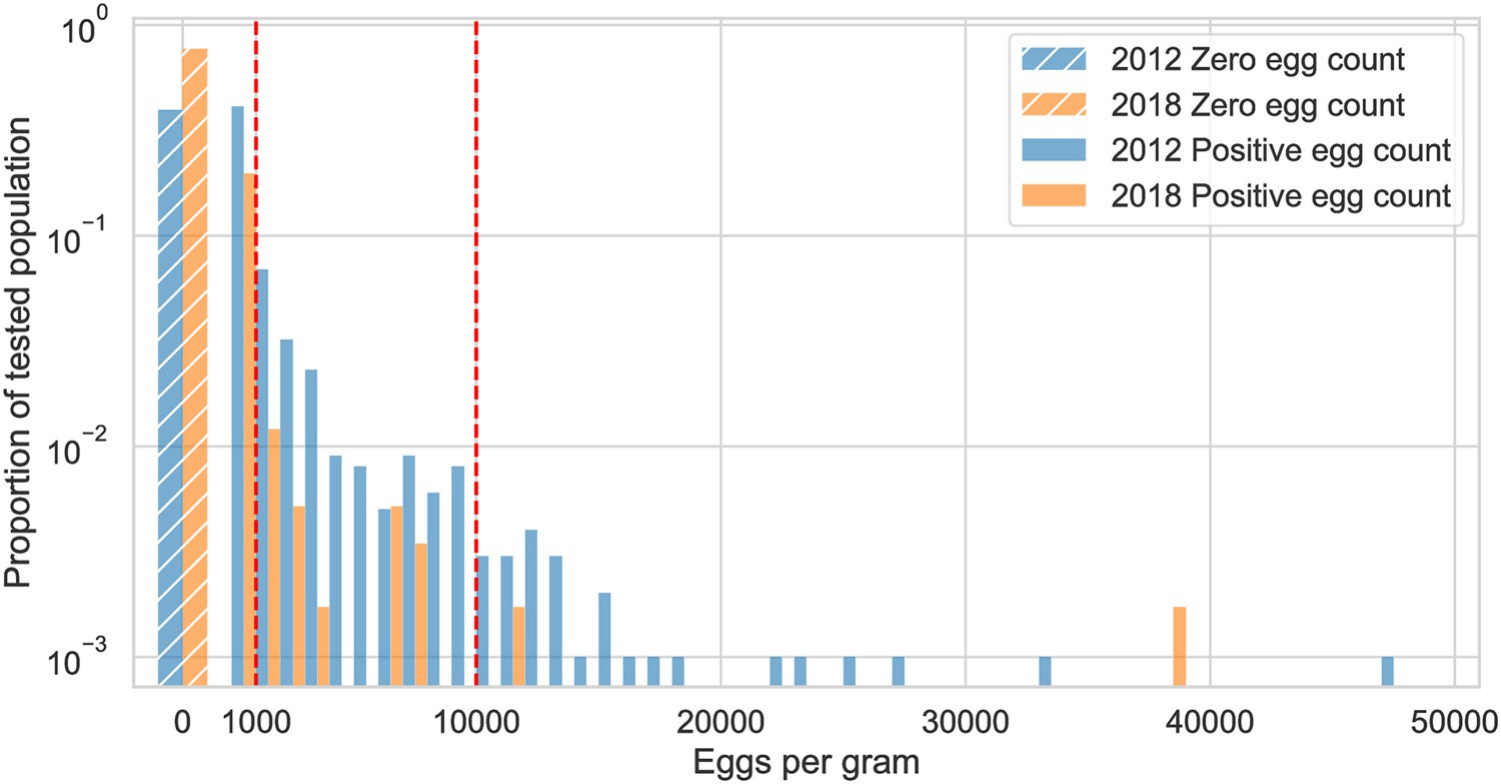
Distribution of EPG among humans in data collected in 2012 and 2018. The hatched wide bars on the left show the proportion of individuals with an egg count of zero. The remaining bars show the proportion of individuals with nonzero egg counts, where the bin width equals 1000 EPG. The red lines mark the thresholds of World Health Organization worm burden classification with 1–999 EPG being classified as light infection, 1000–9999 as moderate infection and 10000 and above as heavy infection [43].

To calibrate the model and intervention parameters, we use previously published data collected in cross-sectional surveys on two adjacent Mekong river islands in Southern Laos in 2012 [14] and previously unpublished data collected from the same villages in 2018. The data from both 2012 and 2018 is included with the publicly available model code. Between the surveys in 2012 and 2018, community-wide MDA was conducted every year except in 2013, community-wide education campaigns were carried out and latrine coverage was increased from 44% to almost 100%. For MDA, praziquantel was distributed at 40mg per kg of body weight and individuals were educated about the risks and prevention of *O. viverrini* infection in the course of each MDA distribution. The data from 2012 and 2018 was collected shortly before the MDA campaigns were carried out in these years. The 2012 data includes the intensity of infection for *O. viverrini* measured in eggs per gram of stool (EPG) in stool samples of humans, cats and dogs. It also includes prevalence of infection in the intermediate snail and fish hosts. For the year 2018, only EPG data in humans is available. The number of samples of each kind are listed in Table 1 and the distribution of EPG among humans is shown in Fig 1. We transform EPG to worm counts and vice versa in individuals and animals with a density-dependent transformation function where the EPG output per worm decreases with an increasing number of worms in an individual [41]. We assume that a single parasite within a host is capable of producing eggs through self-fertilization [42].

**Table 1:** Number of tested and positive samples from definitive and intermediate hosts in 2012 and 2018.

	2012					2018
	Humans	Cats	Dogs	Snails	Fish	Humans
Tested	994	64	68	3102	628	580
Positive	603	34	17	9	169	135
Prevalence	60.7%	53.1%	25%	0.3%	26.9%	23.3%

### 2.2 Agent-based model

**Fig 2:**
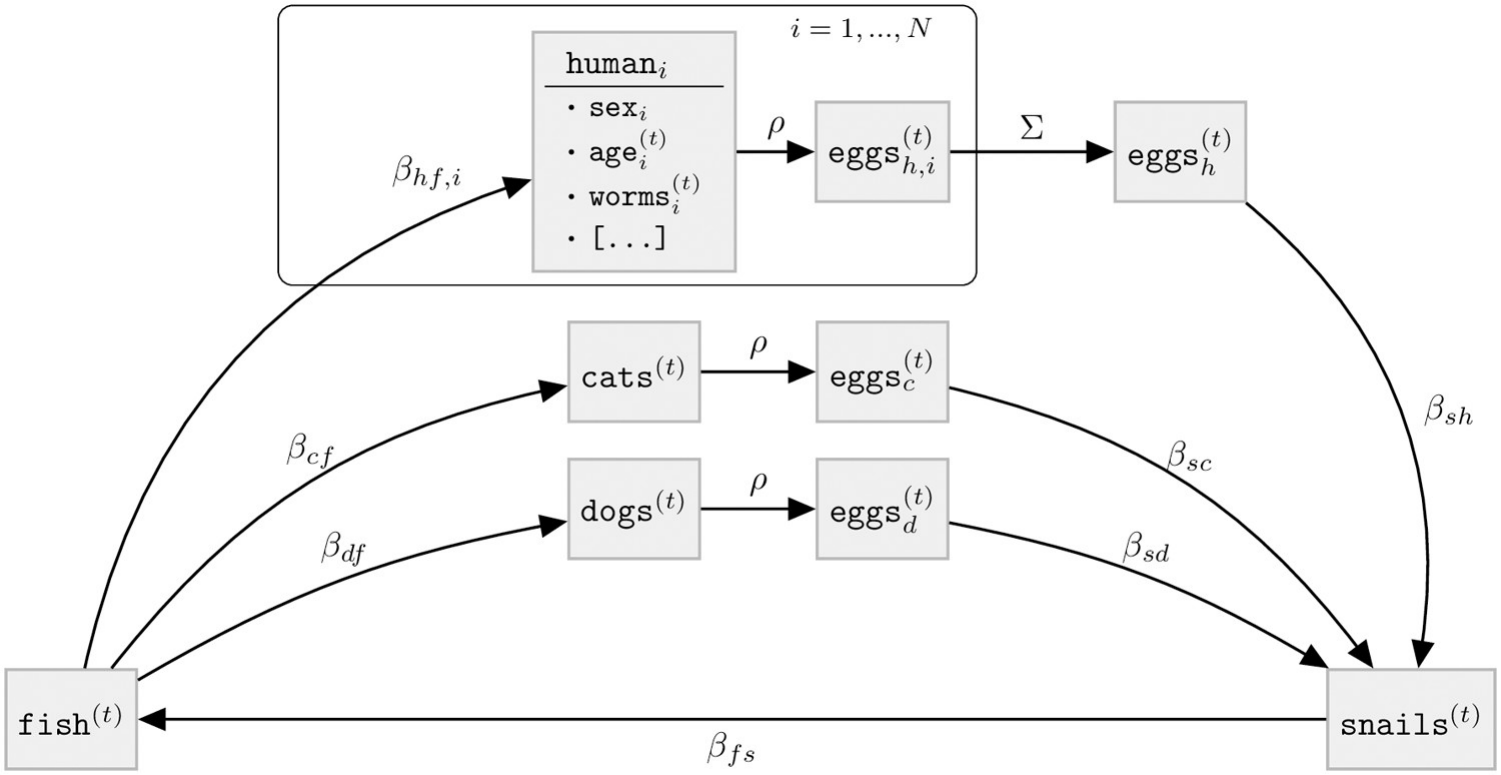
Schematic of the the ABM for *O. viverrini* transmission and control. The state variables are described in Table 2 and the transmission parameters are described in Table 3. EPG output is calculated from worm counts using a density-dependent function *ρ* derived from purging studies (Section A.6.2 in S1 Appendix). While *ρ* is applied to the mean worm burden in cats and dogs and then multiplied with the number of cats and dogs, respectively, it is applied to each individual worm count in humans and subsequently summed up over humans.

This section provides a high-level overview of the ABM (Fig 2). A detailed description written according to the ODD (Overview, Design concepts, Details) protocol for describing individual- and agent-based models [44, 45] is provided in Section A in S1 Appendix. State variables of *N* humans are tracked individually while animals are modeled at the aggregate level (Table 2). The transmission dynamics are governed by the parameters listed in Table 3.

**Table 2:** State variables in the ABM. The value of all variables with superscript (*t*) can change over time. The upper part of the table contains the variables which are tracked for each individual human as indicated by subscript *i*. The lower part of the table contains the state variables of nonhuman hosts. The maximum number of worms an individual can carry is set to a biologically implausible value to facilitate the parameter fitting process and is never reached under fitted parameter values. The number of nonhuman hosts is set relative to the number of modeled humans, *N*.

Variable	Type	Description	Range
sex_*i*_	Boolean	0 for female, 1 for male	{0, 1}
${\text{age}}_{i}^{\left(t\right)}$	Float	Age in days	[0, 100 × 365]
${\text{worms}}_{i}^{\left(t\right)}$	Integer	Number of adult worms in an individual	[0, 10^5^]
${\text{epg}}_{h,i}^{\left(t\right)}$	Integer	Eggs per gram in individual’s stool	[0, *ρ*(10^5^)]
$\text{beta}\_{\text{multiplier}}_{i}^{\left(t\right)}$	Float	Multiplier of *β*_*hf*,*i*_ from education campaign	[0, 1]
${\text{eating}}_{i}^{\left(t\right)}$	Boolean	1 if individual is consuming raw or undercooked fish	{0, 1}
${\text{latrine}}_{i}^{\left(t\right)}$	Boolean	1 if individual has access to a latrine	{0, 1}
dogs^(*t*)^	Integer	Total number of worms in dogs	≥ 0
cats^(*t*)^	Integer	Total number of worms in cats	≥ 0
snails^(*t*)^	Integer	Number of infected snails	[0, *N* × 100]
fish^(*t*)^	Integer	Number of infected fish	[0, *N* × 10]

**Table 3:** Transmission parameters of the ABM. Further parameters of the ABM are described in Section A.6 in S1 Appendix.

Parameter	Description	Unit
*β*_*sh*_	Transmission rate to snails from eggs in human stool	1/Day
*β*_*sd*_	Transmission rate to snails from eggs in dog stool	1/Day
*β*_*sc*_	Transmission rate to snails from eggs in cat stool	1/Day
*β*_*fs*_	Transmission rate to fish from snails	1/Day
*β*_*hf*,*i*_	Transmission rate to individual *i* from fish	1/Day
*β*_*df*_	Transmission rate to dogs from fish	1/Day
*β*_*cf*_	Transmission rate to cats from fish	1/Day

We introduce heterogeneity in worm burden at equilibrium by letting the worm acquiring rate *β*_*hf*,*i*_ vary between individuals, *i*. We assume the *β*_*hf*,*i*_ to follow a gamma distribution, which is a common assumption in ABMs for schistosomiasis [46]. The gamma distribution is parameterized by the mean and variance parameters $\beta_{\mu }^{\Gamma }$ and $\beta_{\sigma^{2}}^{\Gamma }$ and we determine their values by fitting to the data collected in 2012.

For all humans and animals, the uptake of new parasites is Poisson distributed with a mean rate determined by the current state of the system, the transmission parameters, and the time step size. Here, we choose 120 time steps per year, corresponding to a time step size of approximately 3 days, and a population size of 15,000, which roughly corresponds to the population size of the area in which the data was collected. We assume that the population size of cats and dogs is one sixth that of humans, the population size of fish is 10 times that of humans and the population size of snails is 100 times that of humans. The relative population size of cats and dogs is based on a survey conducted among households in the area of data collection [47]. The population sizes of snails and fish are highly speculative but errors in these assumptions will be offset with transmission parameter fitting and will not affect the results presented here.

### 2.3 Population-based model

**Fig 3:**
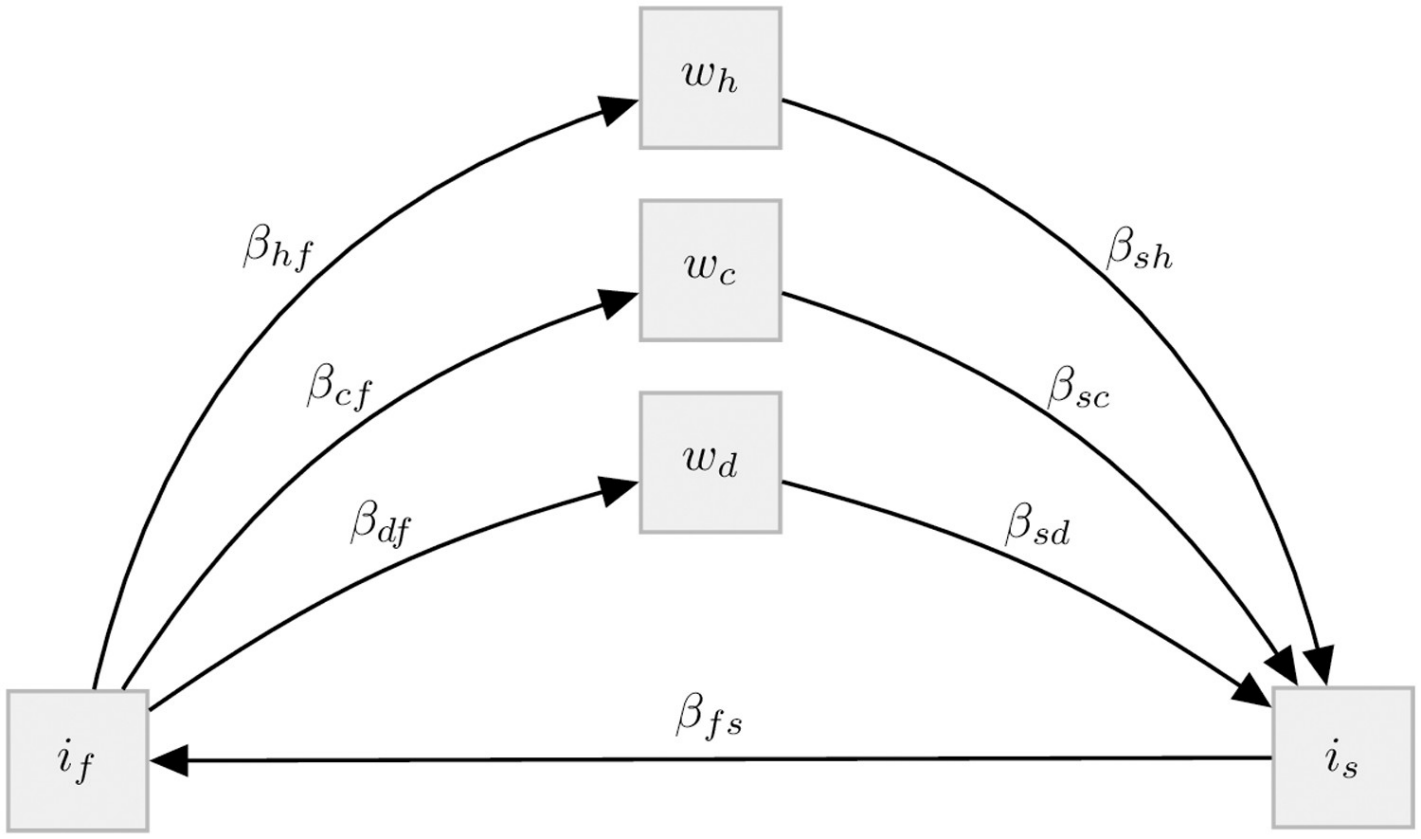
Schematic of the PBM for *O. viverrini* transmission and control. The state variables are described in Table 4, the transmission parameters roughly correspond to the transmission parameters of the ABM listed in Table 3. As opposed to the ABM, where *β*_*hf*,*i*_ varies over individuals, there is only a single *β*_*hf*_ in the PBM. Also, the PBM does not translate worm burden in definitive hosts to egg output which results in the transmission parameters *β*_*sh*_, *β*_*sd*_ and *β*_*sc*_ acting on mean worm burden instead of egg count. Further details on the PBM are provided in Section B in S1 Appendix.

The PBM we use for comparison with the ABM is an ordinary differential equation model that has been described and analysed in previously [31, 32]. A schematic of the model is given in Fig 3 and Table 4. The equations and a complete table of parameters are given in Section B in S1 Appendix.

**Table 4:** State variables of the PBM.

Variable	Description
*w*_*h*_	Mean worm burden per human host
*w*_*d*_	Mean worm burden per dog host
*w*_*c*_	Mean worm burden per cat host
*i*_*s*_	Proportion of infectious snails
*i*_*f*_	Proportion of infectious fish

Instead of tracking worms in humans individually, the PBM tracks the mean number of worms across humans. This means that the PBM, as opposed to the ABM, does not provide a distribution of worm burden and therefore prevalence as a direct output. To obtain a distribution of worms, we use a negative binomial parameterized by an aggregation parameter *k* with the probability mass function,

$$\begin{array}{c} p\left(w_{i}\right)=\frac{(k+w_{i}-1)!}{w_{i}!\left(k-1\right)!}{\left(1+w_{h}/k\right)}^{-k-w_{i}}{\left(w_{h}/k\right)}^{w_{i}}, \end{array}$$ (1)

which provides the distribution of worm counts in individuals, *w*_*i*_, for a given mean worm burden in the population, *w*_*h*_. This aggregation parameter *k* is commonly used in macroparasitic models [15], where a lower value of *k* indicates a higher degree of heterogeneity in the sense that some individuals have a very heavy worm burden compared to the majority of the population. It follows that the prevalence, *P*, which corresponds to the proportion of non-zero worm counts, given *w*_*h*_ and *k* is

$$\begin{array}{c} P=1-{\left(1+w_{h}/k\right)}^{-k}. \end{array}$$ (2)

We substitute the mean worm burden and prevalence from the field data collected in 2012 for *w*_*h*_ and *P* in Eq (2) and numerically solve for *k* to estimate the level of aggregation in the field data. We then assume *k* to be constant over time [15] and substitute the calculated value of *k* into Eq (1) to obtain a distribution of individual worm burden, *w*_*i*_, for any mean worm burden, *w*_*h*_, calculated during a PBM model run.

### 2.4 Transmission parameter fitting

We fit the transmission parameters included in Table 3 to the 2012 data while the 2018 data is not used to fit transmission parameters, but to fit systematic adherence at a later stage. For the ABM, we do not fit the uptake rates of individuals, *β*_*hf*,*i*_, but instead the parameters which govern the distribution of *β*_*hf*,*i*_: $\beta_{\mu }^{\Gamma }$ and $\beta_{\sigma^{2}}^{\Gamma }$, as described in Section 2.2. The parameters governing transmission from definitive hosts to snails, *β*_*sh*_, *β*_*sc*_ and *β*_*sd*_, cannot be uniquely determined given the data for both the PBM and the ABM. We therefore fix these three parameters to be equal to a parameter *β*_*sx*_ which we fit instead. This implies that eggs in stool of cats, dogs and humans that get into the environment are equally likely to infect a snail. However, the ABM takes into account that 44% of humans had a latrine available in 2012 [14], and therefore not all eggs in human stool reach the environment. The transmission parameters are calibrated such that the models reach an equilibrium that matches various target summary statistics calculated from the 2012 data listed in Table 5.

**Table 5:** Target summary statistic values based on 2012 data used to fit transmission parameters.

Summary statistic	Target value
Prevalence humans	60.66%
Mean EPG per human	1108.24
Worms per dog	1.24
Worms per cat	74.12
Prevalence snails	0.29%
Prevalence fish	27.15%

We fit the ABMs using Trust Region Bayesian Optimization (TuRBO) with local Gaussian processes implemented with the Python package BoTorch [48, 49]. The PBM parameters required to reach the target equilibrium were calculated analytically.

### 2.5 Intervention modeling without systematic adherence

We compare the predicted impact of interventions by the PBM and the ABM with the following set of interventions: A yearly MDA campaign with 80% coverage, a one-time education campaign which reduces the consumption of raw fish by 90% without decay, and an increase in latrine coverage from 44% to 90%. This approximates the interventions that were implemented between the dates of field data collection in 2012 and 2018 (Section 2.1). We chose an 80% MDA coverage, as this was estimated to be the effective coverage by expert opinion. We choose a mean reduction of 90% in fish consumption by the education campaign for better comparison as this is the mean estimated reduction in models that include systematic adherence. While the reported latrine coverage was increased to 100%, we assume that not all individuals always use it and that some collected feces is used as fertilizer, though the choice of 90% for effective latrine coverage is arbitrary.

For the PBM, the interventions are implemented as follows: The MDA leads to a 80% reduction in mean worm burden each time it is deployed. The education campaign results in a reduction of the transmission parameter from fish to humans, *β*_*hf*_, by 90%. The increase in latrine coverage from 44% to 90% is implemented by a reduction in *β*_*sh*_ by 82%, as *β*_*sh*_ in the PBMs was estimated without explicitly modeling latrines. For the ABM, MDA is modeled by setting the worm count to 0 for 80% of the population randomly selected each time an MDA campaign is conducted. The education campaign is implemented by setting the $\text{beta}\_{\text{multiplier}}_{i}^{\left(t\right)}$ to 0.1 for all individuals. This will result in a 90% reduction in the mean uptake of new worms for all individuals, as each individual’s transmission parameter, *β*_*hf*,*i*_, is multiplied with this individual’s $\text{beta}\_{\text{multiplier}}_{i}^{\left(t\right)}$ in order to determine the individual’s worm uptake rate in each time step (Sections A.7.2 and A.7.4 in S1 Appendix). The increase in latrine coverage is modeled by randomly assigning latrines to individuals which do no have a latrine yet such that the total coverage is 90%. We run the simulations for the ABM with 10 random seeds.

We start both models at equilibrium, run them for one year in equilibrium before deploying the interventions, where the MDA is implemented every year for five years. We then compare the model predictions to the field data collected in 2018 one year after the last MDA campaign.

### 2.6 Intervention modeling with systematic adherence

We introduce systematic adherence in the ABM for the MDA and the education campaign in order reproduce the data collected in 2018 after the implementation of interventions. We assume that the systematic adherence is correlated with individuals’ worm acquisition rates *β*_*hf*,*i*_ as described in the following two paragraphs.

For the MDA, we assume that the coverage of the MDA campaign stays as 80% over the entire population but that each individual is assigned a random probability of adhering to the MDA campaign, *p*_*m*,*i*_, which stays fixed over time (Section A.7.1 in S1 Appendix). We assume *p*_*m*,*i*_ to follow a beta distribution with mean *μ*_*m*_ = 0.8 and variance $\sigma_{m}^{2}$, where $\sigma_{m}^{2}$ will be estimated. We introduce negative correlation between *β*_*hf*,*i*_ and *p*_*m*,*i*_ by drawing these variables from a Gaussian copula with a beta and a gamma marginal distribution (for the *p*_*m*,*i*_ and *β*_*hf*,*i*_ respectively) and a correlation of -0.9 between the latent Gaussian variables. This will result in individuals that have a higher worm acquisition rate *β*_*hf*,*i*_ being less likely to adhere to an MDA campaign. This mechanism introduces serial correlation in individuals’ adherence over multiple rounds of MDA, or, in other words, systematic adherence.

For the education campaign, we introduce systematic adherence by introducing an individual-specific education campaign factor *e*_*i*_, where with each education campaign, the $\text{beta}\_{\text{multiplier}}_{i}^{\left(t\right)}$ of individual *i* gets multiplied with *e*_*i*_ (Section A.7.2 in S1 Appendix). We assume *e*_*i*_ to follow a beta distribution with mean *μ*_*e*_ and variance $\sigma_{e}^{2}$, both of which will be estimated. We introduce positive correlation between *β*_*hf*,*i*_ and *e*_*i*_ by drawing these variables from a Gaussian copula with a beta distribution and a gamma distribution as marginals (for the *e*_*i*_ and *β*_*hf*,*i*_ respectively) and a correlation of 0.9 between the latent Gaussian variables. This will result in individuals that have a higher worm acquisition rate *β*_*hf*,*i*_ having a higher education campaign factor *e*_*i*_, translating to a smaller effect of the education campaign on their consumption of raw or undercooked fish. We interpret this different effect of the education campaign on individuals, which stays fixed over time for each individual, as systematic adherence to the education campaign.

We now model education campaigns to take place every year together with the MDA. We make this choice in order to be able to predict the impact of the deployed interventions into the future beyond the year of final measurement in 2018, assuming that education campaigns would continue being conducted after the study period. We therefore also include waning of the effects of the education campaign by letting $\text{beta}\_{\text{multiplier}}_{i}^{\left(t\right)}$ approach 1 in an exponential-like manner at each time step (Section A.7.2 in S1 Appendix). Together with the regularly conducted education campaigns, this leads to $\text{beta}\_{\text{multiplier}}_{i}^{\left(t\right)}$ approaching a periodic function in the long run.

This setup leaves us with 3 parameters to estimate in order to reproduce of the 2018 field data starting from an equilibrium reproducing the 2012 field data: The variance $\sigma_{m}^{2}$ in the distribution over MDA adherence probabilities, *p*_*m*,*i*_; and the mean *μ*_*e*_ and the variance $\sigma_{e}^{2}$ for the distribution of education campaign factors, *e*_*i*_. The two variance parameters, $\sigma_{m}^{2}$ and $\sigma_{e}^{2}$, estimate the degree of systematic adherence required to explain the 2018 data.

We run simulations for one year in equilibrium followed by an increase in latrine coverage from 44% to 90% as well as yearly MDA and education campaigns for various parameter values for $\sigma_{m}^{2}$, *μ*_*e*_, and $\sigma_{e}^{2}$. We compare the mean worm burden, overall prevalence, prevalence of moderate worm burden and prevalence of heavy worm burden predicted by the model for 2018 to the data collected in 2018 (Table 6). Using a Gaussian process regression emulator over the space of the three parameters, we find the combination of parameters $\sigma_{m}^{2}$, *μ*_*e*_, and $\sigma_{e}^{2}$ which most closely reproduces the 2018 data. We conduct a global Sobol sensitivity analysis over the parameter space using the python library SALib [50, 51] in order to determine which of the parameters were essential for the reproduction of which summary statistic of the 2018 data.

**Table 6:** Target summary statistic values based on 2018 data used to fit systematic adherence and the mean effect of the education campaign.

Summary statistic	Target value
Mean worms per human	75.79
Prevalence humans	22.76%
Prevalence heavy worm burden	0.34%
Prevalence moderate worm burden	3.03%

We also predict the time it takes to achieve a reduction in prevalence of heavy worm burden below 1/10,000 and of overall prevalence below 1% with the fitted systematic adherence. For this, we continue the interventions deployed between 2012 and 2018 for a total of 80 years. Concretely, we continue yearly MDA and education campaigns, whereas the increase in latrines stays a one-time intervention that is only deployed once in 2013. We disentangle the effect of systematic adherence by comparing the predicted times to reach the targets under systematic adherence and in the absence of systematic adherence. We also test the time it would have taken if interventions with the fitted properties were deployed in isolation in order to assess their individual contributions to achieve the target reduction. We consider reaching a prevalence of heavy worm burden below 0.01% as a substantial reduction of the public health burden, analogous to targets defined for other NTDs [52].

### 2.7 Model variant with only humans as definitive hosts

We also ran all analyses described above for a variant of the models where we fix the transmission from fish to cats and dogs to be zero, which removes these hosts from the transmission cycle. This model was created to test the impact of including cats and dogs as definitive hosts, since there is uncertainty on whether humans and other mammals are infected by the same population of *O. viverrini* parasites [53].

## 3 Results

### 3.1 Equilibrium fit

**Fig 4:**
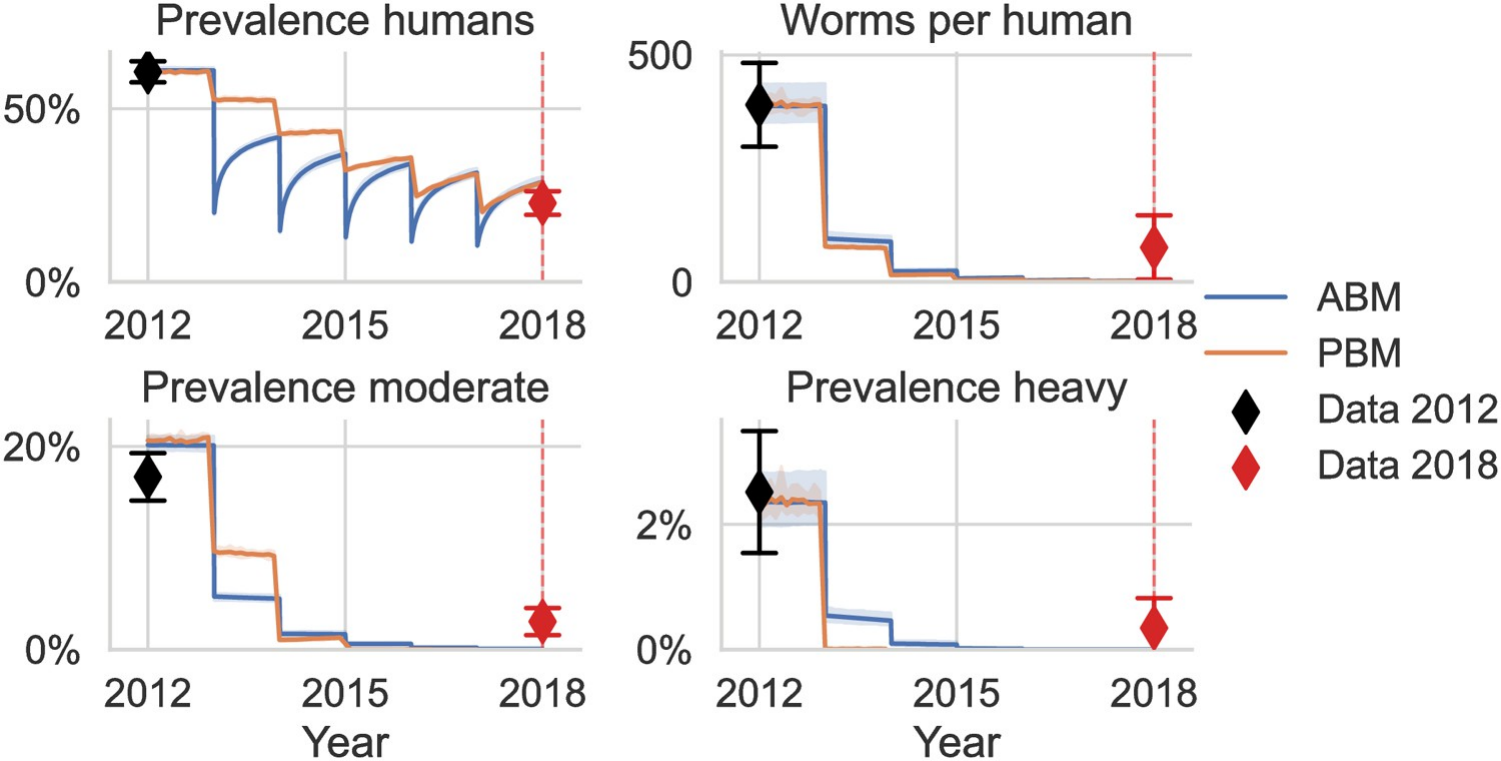
Time series for prevalence, mean worm burden, prevalence of moderate worm burden and prevalence of heavy worm burden as predicted by the models starting in equilibrium followed by five years of interventions without systematic adherence (MDA, education campaign, improved sanitation). Both models were calibrated to fit the 2012 data in equilibrium but not the 2018 data. Additional variables of the model runs are provided in Fig I in S1 Appendix.

**Fig 5:**
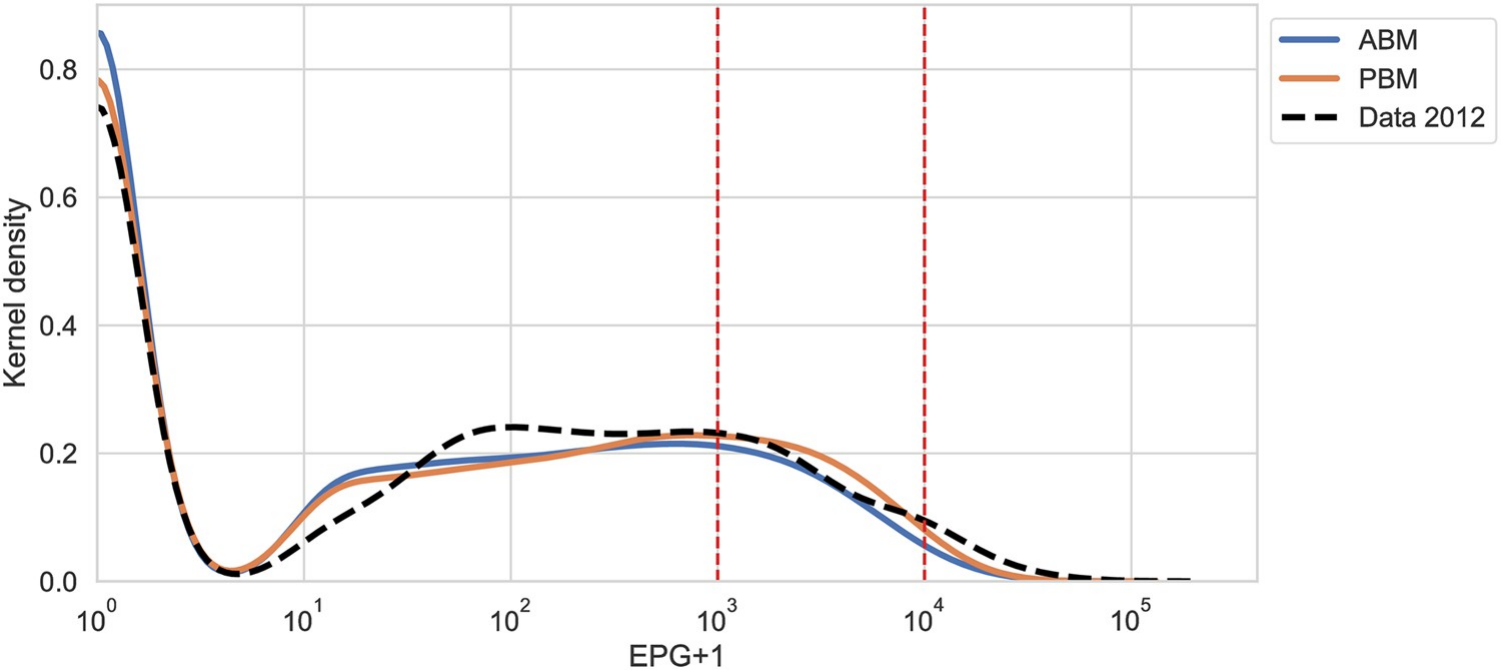
Kernel density estimate of EPG in the 2012 data and in the equilibrium state of the ABM and the PBM. The x-axis is plotted on a log scale. The dashed vertical red lines indicate the transition from light to moderate and from moderate to heavy worm burden, respectively.

Both the ABM and PBM reproduce the distribution of worm burden observed in 2012 reasonably well in equilibrium with the fitted parameter values (Table 7). The equilibrium fit can be visually assessed by comparing the model equilibrium values of mean worm burden and prevalence to the data collected in 2012 (Fig 4 and Fig I in S1 Appendix), as well as by comparing the kernel density estimate of the modeled egg per gram output to that of the data collected in 2012 (Fig 5). Remarkably, even though only the mean worm burden and prevalence were used as a fitting objectives, both models fit the prevalence of light, medium, and heavy worm burden, as well as the mean worm burden within each of these groups very closely. The distribution in EPG in equilibrium between the ABM and the PBM is very similar, which can be expected: draws from a Poisson distribution with rates that are gamma-distributed follow a negative binomial distribution; in the ABM, the worm uptake of individuals in a time step follows a Poisson distribution with uptake rates *β*_*hf*,*i*_ that are gamma-distributed over individuals.

**Table 7:** Fitted parameter values for the PBM and the ABM. The fitted values for *β*_*df*_, *β*_*cf*_ and *β*_*fs*_ are very close for both models. The fitted values for *β*_*sx*_ differs between the ABM and the PBM since *β*_*sx*_ acts on the total egg output in the ABM whereas it acts on mean worm burden in the PBM.

Parameter	ABM	PBM
$\beta_{\mu }^{\Gamma }$	0.485	–
$\beta_{\sigma^{2}}^{\Gamma }$	1.986	–
*β*_*hf*_	–	0.394
*β*_*df*_	0.003	0.003
*β*_*cf*_	0.189	0.187
*β*_*sx*_	1.275 × 10^−10^	1.712 × 10^−8^
*β*_*fs*_	0.141	0.141

### 3.2 Intervention modeling without systematic adherence

The ABM and the PBM are also similar in their prediction of the impact of the intervention combination of MDA, education, and improved sanitation (Fig 4). The predicted drops in prevalence that immediately follow MDA distribution campaigns are smaller for the PBM than for the ABMs, implying a higher degree of aggregation in the ABM following immediately after the distribution of the drugs. With the PBM, the prevalence of individuals with heavy worm burden drops to almost 0 after the first MDA campaign, while there is more persistence of heavy worm burden with the ABM.

Comparing the intervention impact predictions of the models for 2018 with the data collected in 2018, there is a slight overestimation of prevalence and a much lower predicted mean worm burden (Fig 4). The underestimation in mean worm burden is a consequence of the quick drop and subsequent underestimation in prevalence of individuals with moderate and heavy worm burden. If each individual has a probability of 80% of adhering to the MDA, the probability of not adhering over five rounds is 0.03%. Therefore, most individuals, including those with heavy worm burden, get cleared of all parasites at least once over the MDA campaigns and the estimated infection rates are not high enough for heavy worm burden to reoccur in the short time until the next MDA campaign is conducted.

### 3.3 Intervention modeling with systematic adherence

**Fig 6:**
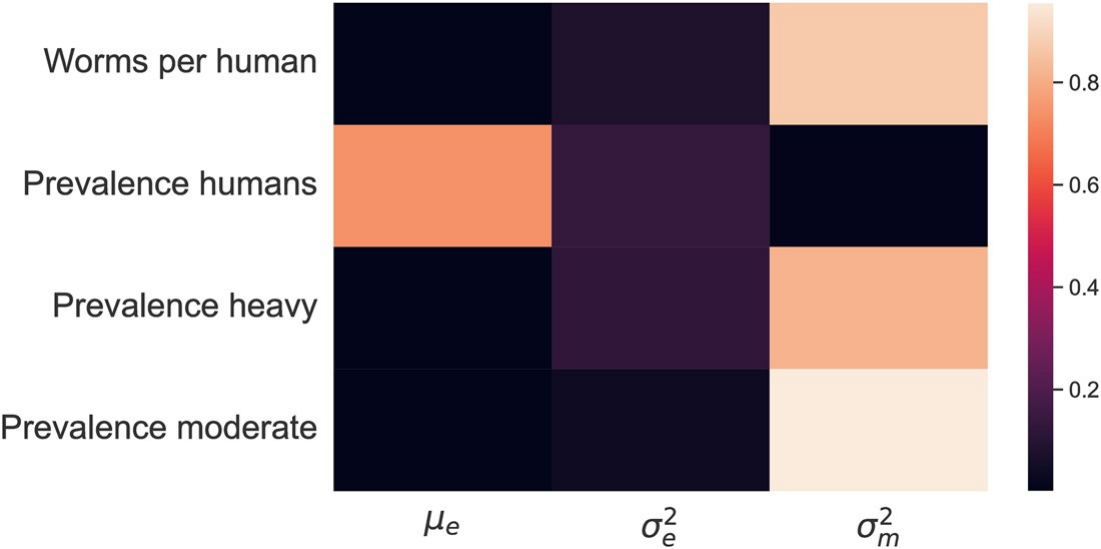
First-order Sobol indices measured in the global sensitivity analysis of fitted intervention parameters (x-axis) on goodness of fit of the model to the objective summary statistics in the 2018 data (y-axis, Table 7). The indices sum up to one in each row. The parameter controlling the mean effect of the education campaign, *μ*_*e*_, has mostly a strong impact on the prevalence in humans. Systematic adherence to the education campaign, controlled through the parameter $\sigma_{e}^{2}$, affects prevalence, prevalence of heavy worm burden, and mean worm burden, though for each one of these outcomes, one of the other two studied parameters is of greater importance. Systematic adherence to the MDA campaign, controlled through the parameter $\sigma_{m}^{2}$, has a strong effect on the prevalence of moderate and heavy worm worm burden and consequently on the mean worm burden in the population.

The estimated values for $\sigma_{m}^{2}$, *μ*_*e*_, and $\sigma_{e}^{2}$ are 0.032, 0.19 and 0.084 respectively. The estimated value for $\sigma_{m}^{2}$ corresponds to an odds ratio of 2.97 for adherence given previous adherence. The sensitivity analysis shows that the persistence of mean worm burden depends on the systematic adherence to MDA through persistence of individuals with moderate and heavy worm burden (Fig 6). On the other hand, overall prevalence is mainly driven by the mean effect of the education campaign, while the extent of systematic adherence to the education campaign plays a lesser role.

**Fig 7:**
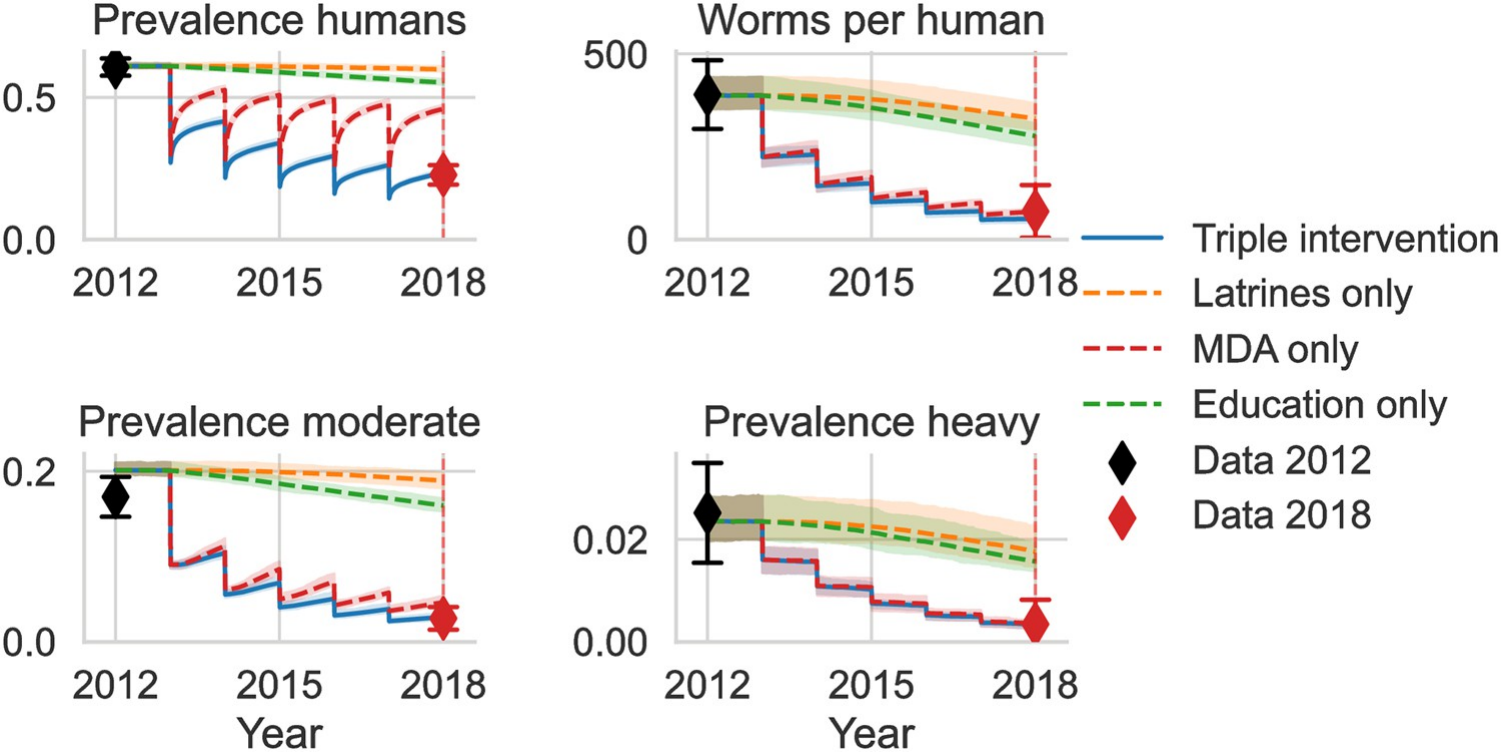
Time series for the ABM with fitted systematic adherence where all three interventions are implemented together or in isolation. Additional variables of the model runs are provided in Fig I in S1 Appendix.

The model with the fitted systematic adherence to interventions closely reproduces the 2018 target summary statistics (Fig 7). When implementing each of the interventions separately, the results of the sensitivity analysis are reflected in the simulated time series: MDA alone can drastically reduce the mean worm burden, while the predicted reduction is much smaller for latrines or education alone over this limited time period. This is also true for the prevalence of moderate and heavy worm burden. What is not obvious from the presented first-order sensitivity analysis, is that education alone is not does not substantially reduce the prevalence over this period of 5 years. It is only in combination with MDA that an education campaign strongly reduces overall prevalence. This can be explained by the prevention of reinfection through education after the MDA campaigns. Persistent education campaigns in isolation can still reduce prevalence when implemented over a longer time period, as shown in the next section. We predict latrines to have the smallest impact over this period of 5 years on mean worm burden and prevalence when deployed in isolation.

Systematic adherence increases the time it takes for interventions to achieve the public health goals (Table 8). This is true when interventions are combined together or deployed in isolation. We predict that MDA alone reduces heavy worm burden prevalence below 1/10,000 as quickly as when it is combined with latrines and education, whether we assume systematic adherence or not. We predict education campaigns only and latrines only also to reduce heavy worm burden prevalence below 1/10,000, although we predict this to take longer than with MDA alone. The reduction in overall prevalence below 1% can be achieved with MDA alone as well, even under systematic adherence. As opposed to the reduction in heavy worm burden prevalence, there is a clear benefit of combining interventions in terms of the time it takes to achieve the reduction goal for overall prevalence. We predict education alone to take very long to achieve the overall prevalence target if there is no systematic adherence and to not achieve it within 80 years if there is systematic adherence. We also predict latrines alone not to be sufficient to reduce overall prevalence below 1% within 80 years.

**Table 8:** Year in which two public health targets using all interventions together or in isolation, and with (SA) or without assuming systematic adherence (NSA), are met. The targets are reducing the prevalence heavy worm burden below 1/10,000 and reducing overall prevalence below 1%. The standard errors of the mean in years over the simulations with the 10 random seeds are given in brackets. Latrines are only modeled without systematic adherence, and hence the analysis is not applicable (NA) for latrines only with systematic adherence. The reduction of prevalence below 1% cannot be achieved within 80 years with education under systematic adherence or with latrines only.

	Heavy < 0.01%		Prevalence < 1%	
	NSA	SA	NSA	SA
Triple intervention	2016 (0.22)	2029 (1.11)	2028 (0.13)	2057 (0.87)
MDA only	2017 (0.15)	2028 (0.57)	2051 (0.74)	2071 (1.96)
Education only	2029 (0.52)	2038 (0.72)	2082 (0.59)	–
Latrines only	2040 (1.23)	NA	–	NA

### 3.4 Model variant with only humans as definitive hosts

The qualitative results and conclusions are not affected by excluding cats and dogs from the transmission cycle (Section D in S1 Appendix). The removal of cats and dogs leads to a higher estimated transmission rate from humans to snails, *β*_*sh*_, and to a slightly faster reduction in worm burden by the modeled interventions.

## 4 Discussion

We present a new ABM for *O. viverrini* transmission and control and show that, in the absence of systematic adherence, the model predictions of the ABM are very similar to those made by a previously published ordinary differential equation model. We then demonstrate how adding systematic adherence to MDA and education campaigns in the ABM can explain changes in the distribution of worm burden observed in field data after a five year intervention period that consisted of MDA and education campaigns, as well as an increase in latrine coverage. When focusing on the dynamics during the 5 years in which the interventions were implemented, we show how systematic MDA adherence is a plausible cause for the observed persistence of heavy worm burden in some individuals. On the other hand we show that education campaigns, and therefore behavior change, in combination with MDA are a plausible cause for the observed reduction in overall prevalence of infection.

Predicting the impact of interventions into the future and focusing on heavy worm burden, we show how systematic adherence to MDA can substantially increase the time it takes to strongly reduce the prevalence of heavy worm burden. However, even if only MDA is deployed and high-risk individuals do systematically miss MDA campaigns, the model predicts that MDA alone can strongly reduce the prevalence of heavy worm burden due to the reduction of worm burden in the general population and the associated reduction of parasites in intermediate hosts. This might differ from other parasitic diseases, where localized pockets of infections play a more important role for persistence of heavy infections [30]. This result can be explained by the assumption of homogenous mixing of fish and humans, which is a reasonable assumption at the relatively small geographical scale of the study setting given the mobility of fish [54]. While this finding highlights the benefits of MDA even under systematic adherence, the much quicker reductions achieved under non-systematic adherence highlight the potential benefit of reaching individuals that tend to systematically miss MDA campaigns. Besides MDA, we also predict the education campaign to strongly reduce heavy worm burden, though slower than with MDA. We also note that there is more uncertainty regarding the true effectiveness of the education campaign as we estimated both its effect size and its variance and assumed that the education campaign results in a reduction of raw fish consumption every year.

Predicting the impact of interventions into the future and focusing on overall prevalence, we show how adding education campaigns and latrines to MDA campaign can drastically reduce the time it takes to achieve a prevalence below 1%. This highlights the importance of combining interventions for the reduction of people infected with *O. viverrini*. However, with the estimated systematic adherence, even with triple interventions, we predict it takes more than 40 years of interventions in order to reduce overall prevalence below 1%. Without systematic adherence, this goal is achieved substantially quicker. Again, this results highlights the importance of breaking patterns of systematic adherence.

The work presented here also adds to the broader body of research on modeling of systematic adherence. It is to our knowledge the first time that the extent of systematic adherence is estimated from epidemiological data with a mathematical model instead of directly assessing it through observations or surveys. While this indirect approach can end up attributing effects to systematic adherence that are in fact caused by other mechanics, it does not require the direct collection of data on systematic adherence, which can be very costly [24]. The estimated odds ratio of 2.97 for adherence given past adherence lies within the range of estimated quantities from empirical studies.

There are several important factors we have not considered for simplicity or because of data unavailability. MDA campaigns have been conducted in the study area prior to 2012, albeit on an irregular basis [14] and we lack data on the timing and the coverage of these campaigns. The assumption of an equilibrium at the start of the simulation period may therefore be inappropriate and yield parameter fits which underestimate transmission intensities.

We have also not considered inaccuracies of the EPG measurement technique. Specifically, sensitivity may not be perfect, leading to an underestimation of prevalence, and the EPG counts for heavy worm burden may be subject to large variances. We also assumed MDA to have perfect efficacy, leading to clearance of all parasites when an individual receives the drug. This assumption is not realistic with the distributed doses, especially in individuals with heavy worm burden [17]. However, as most individuals are reached by multiple MDA campaigns, it might be reasonable to assume that they are eventually cleared of the parasite. Our modeled approach of continued targeting of the entire population with MDA and education campaigns even after prevalence has been substantially reduced is also not realistic if other diseases targeted by the MDA campaigns are also under control. However, the ABM presented here is well suited to study alternative strategies and potential risks of reducing intervention coverage in the future. In this context, it is also important to note that the estimated times when public health goals are reached should not be understood as predictions. Rather, the estimated years allow us to draw qualitative conclusions about the different impact of interventions and of systematic adherence. We assumed that humans, cats, and dogs are infected by the same population of *O. viverrini* parasites. While this is a common assumption, there is limited evidence in its support [2, 53]. We therefore ran all the simulations assuming no transmission to and from cats and dogs and our qualitative results are not affected by the change of this assumption. Even though we have sample sizes of several hundred individuals, the uncertainty for the proportion of heavy worm burden individuals is high as only very few individuals fall into this category. Specifically, for the year 2012 there were 25 individuals with heavy worm burden, while in 2018, there were only two individuals. While the ABM allows setting a lot of parameters at the level of individuals, we often lack information on how to specify these variables. For example, the choice of a beta distribution for the MDA adherence was chosen for simplicity and may not reflect reality. Finally, our analysis does not include parameter uncertainty for transmission parameters and the estimated interventions properties.

A next step is to integrate the ABM with systematic adherence with a disease model, since ABMs can track current and past worm burden at the individual level. This allows us predict to disease caused by infection with *O. viverrini* and ultimately express the effect of interventions in terms of disability-adjusted life years instead of choosing arbitrary public health goals. Concretely, the disease model could predict incidence of cholangiocarcinoma and other disease based on current and past intensity of infection, treatment history [55] and additional risk factors, such as age. Currently ongoing studies associating worm burden and disease will support the appropriate parameterization of such a disease model.

## Supporting information

S1 Appendix Model description and additional figures. (PDF)
