# Supplementary material for: Modeling the persistence of Opisthorchis viverrini worm burden after mass-drug administration and education campaigns with systematic adherence
Source: PLoS Negl Trop Dis. 2024 Feb 29;18(2):e0011362. doi: 10.1371/journal.pntd.0011362 (PMC10931503; doi:10.1371/journal.pntd.0011362)
Supplement: S1 Appendix — (PDF) [file pntd.0011362.s001.pdf]

S1 Appendix: Model description and additional figures

Modeling the persistence of *Opisthorchis viverrini* worm burden after mass-drug administration and education campaigns with systematic adherence

Lars Kamber[1,2,*], Christine Bürli[1,2], Helmut Harbrecht[3], Peter Odermatt[1,2], Somphou Sayasone[1,2,4], and Nakul Chitnis[1,2]

[1]Swiss Tropical and Public Health Institute, Allschwil, Switzerland
[2]University of Basel, Basel, Switzerland
[3]Department of Mathematics and Computer Science, University of Basel, Basel, Switzerland
[4]Lao Tropical and Public Health Institute, Vientiane, Lao PDR
[*]Corresponding author. Email: lars.kamber@swisstph.ch

A Overview, Design concepts, Details (ODD) model description

This model description follows the Overview, Design concepts, Details (ODD) protocol for describing individual- and agent-based models [1, 2].

A.1 Purpose and patterns

The purpose of this agent-based model (ABM) is to provide impact estimates of various interventions on transmission of *Opisthorchis viverrini* and disease burden caused by *O. viverrini* at the level of a single community. Using these estimates, we aim to provide policy recommendations for disease burden control and elimination. In order to provide useful estimates, the model must reproduce patterns observed in field data, specifically the parasite burden in definitive and intermediate hosts and the heterogeneity of parasite burden and effects of treatment on humans.

A.2 Entities, state variables and scales

Fig A shows a schematic of the entities contained in the ABM. There are `N` human individuals and the environment consisting of reservoir hosts that contribute to the transmission of *O. viverrini.* We choose to model worm burden in reservoir hosts at an aggregate level because the scientific questions we want to answer with the model revolve around the heterogeneity of worm burden in humans, because of limited data availability on reservoir host worm burden and to reduce computational costs. The state variables associated with the entities are listed in Table B.

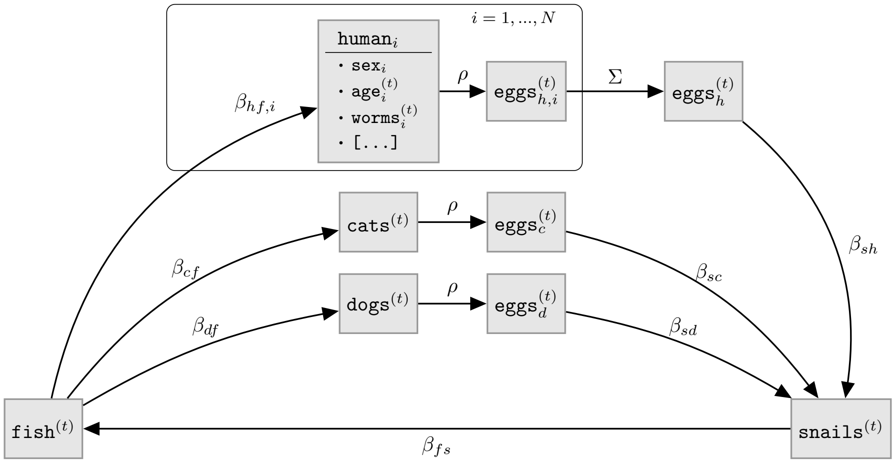

Fig A. Schematic of the ABM.

Variable	Type	Description	Range
`sex`$_i$	Boolean	0 for female, 1 for male	$\{0, 1\}$
`age`$_i^{(t)}$	Float	Age in days	$[0, 100 \times 365]$
`worms`$_i^{(t)}$	Integer	Number of adult worms in individual	$[0, \texttt{worms\_max}]$
`epg`$_i^{(t)}$	Integer	Eggs per gram in individual's stool	$[0, \rho(\texttt{worms\_max})]$
`beta_multiplier`$_i^{(t)}$	Float	Multiplier to $\beta_{hf,i}$ from education campaign	$[0, 1]$
`eating`$_i^{(t)}$	Boolean	Individual is consuming undercooked fish	$\{0, 1\}$
`latrine`$_i^{(t)}$	Boolean	Individual has access to a latrine	$\{0, 1\}$
`latrine_use`$_i^{(t)}$	Boolean	Individual uses latrine if accessible	$\{0, 1\}$
`MDA_treatments`$_i^{(t)}$	Integer	Number of MDA treatments an individual has received	≥ 0
`worm_days`$_i^{(t)}$	Integer	Number of worm days an individual accumulated over lifetime	≥ 0
`dogs`$^{(t)}$	Integer	Total number of worms in dogs	≥ 0
`cats`$^{(t)}$	Integer	Total number of worms in cats	≥ 0
`snails`$^{(t)}$	Integer	Number of infected snails	$[0, N_{snails}]$
`fish`$^{(t)}$	Integer	Number of infected fish	$[0, N_{fish}]$

Table B. State variables tracked in the ABM. The time t at which a variable is measured is denoted by a superscript (t) for variables that change over time, the individual i for which a variable is measured is indicated by a subscript if applicable.

The dynamics of the model are governed by various transmission parameters β which are also depicted in Fig A and listed in Table C.

Spatial factors are not included in the model, as the scope is restricted to a single

Parameter	Description	Unit
β_{sh}	Transmission rate to snails from eggs in human stool	1/(Day × Animals)
β_{sd}	Transmission rate to snails from eggs in dog stool	1/(Day × Animals)
β_{sc}	Transmission rate to snails from eggs in cat stool	1/(Day × Animals)
β_{fs}	Transmission rate to fish from snails	1/(Day × Animals)
$\beta_{hf,i}$	Transmission rate to individual i from fish	1/(Day × Animals)
β_{df}	Transmission rate to dogs from fish	1/(Day × Animals)
β_{cf}	Transmission rate to cats from fish	1/(Day × Animals)

Table C. Transmission parameters of the ABM. The function of the parameters is described in more detail in Section A.7. Parameter values are determined by fitting to field data, as described in the main text.

community and we assume the proximity between humans at a small scale to be irrelevant for transmission given the life cycle of *O. viverrini*. Time t is measured in days. The time step size dt is constant over a simulation run is calculated by dividing 365 by the input parameter `timesteps_per_year`. We set `timesteps_per_year` to 120 for analyses in the main paper resulting in a dt of 3.042 days. The model does currently not support a time step smaller than one day.

A.3 Process overview and scheduling

Submodules are executed in the order listed below at each time step t to calculate the value of the state variables at time $t + dt$. This section provides an overview of the submodules with further details on each submodule given in Section A.7 and a description of parameters given in Tables D and E.

1. "MDA submodule": Checks if an MDA round is scheduled within the time interval $[t, t + dt)$. If this is the case, MDA is distributed according to the scheme specified by the parameter `MDA_plan` and `MDA_coverage` among non-pregnant individuals aged over `MDA_minimum_age`. For individuals receiving MDA, $\texttt{worms}_i^{(t)}$ is set to 0 with an immediate effect on transmission.

2. "Education submodule": Checks if an education round is scheduled within the time interval $[t, t + dt)$. If this is the case, eating education is assigned according to the scheme specified by the parameter `education_strategy` and `education_coverage` among individuals aged over `education_minimum_age`. For individuals receiving education, $\texttt{beta\_multiplier}_i^{(t)}$ is multiplied as specified for the current education round with an immediate effect on transmission.

3. "Latrine submodule": Checks if a change in latrine coverage is scheduled within the time interval $[t, t + dt)$. If this is the case, latrine coverage is changed according the scheme specified by the parameter `latrine_change_coverage`.

4. "Human worms submodule": Calculates $\texttt{worms}_i^{(t+dt)}$ for each individual at time $t + dt$. Subtracts a random number of worms from $\texttt{worms}_i^{(t)}$ for each individual depending on the worm mortality parameter μ_{ph}. Adds a random number of worms to $\texttt{worms}_i^{(t)}$ for each individual depending on the number of currently infected fish $\texttt{fish}^{(t)}$ and the individuals' worm acquiring rate $\beta_{hf,i}$.

5. “Demography submodule”: Assigns mortality rates to each individual depending on variables $\texttt{age}_i^{(t)}$ and $\texttt{sex}_i$. Adds time step size dt to each $\texttt{age}_i^{(t)}$ to calculate $\texttt{age}_i^{(t+dt)}$. Randomly assigns death according to the assigned mortality rates. Replaces individuals that were assigned death with newborn individuals by resetting these individuals' state variables $\texttt{age}_i^{(t+dt)}$ and $\texttt{worms}_i^{(t+dt)}$ to 0, $\texttt{eating}_i^{(t+dt)}$ to `False`, and $\texttt{beta\_multiplier}_i^{(t+dt)}$ to 1.

6. “Reservoir hosts submodule”: Calculates $\texttt{cats}^{(t+dt)}$ and $\texttt{dogs}^{(t+dt)}$. Subtracts random number of worms from $\texttt{cats}^{(t)}$ and $\texttt{dogs}^{(t)}$ according to parasite mortality rates μ_{pc} and μ_{pd}. Adds random number of worms to $\texttt{cats}^{(t)}$ and $\texttt{dogs}^{(t)}$ depending on number of currently infected fish $\texttt{fish}^{(t)}$ and animal worm acquiring rates β_{cf} and β_{df}.

7. “Intermediate hosts submodule”: Calculates $\texttt{snails}^{(t+dt)}$ and $\texttt{fish}^{(t+dt)}$. Calculates the eggs per gram as a nonlinear function of worms for all definitive hosts. Adds random number of infected snails to $\texttt{snails}^{(t)}$ depending on the sum of calculated eggs per gram (EPG) over definitive hosts and transmission rates β_{sh}, β_{sc} and β_{sd}. Subtracts random number of infected snails from $\texttt{snails}^{(t)}$ according to snail mortality rate μ_s. Adds random number of infected fish to $\texttt{fish}^{(t)}$ depending on number of infected snails $\texttt{snails}^{(t)}$ and transmission parameter β_{fs}. Subtracts random number of infected fish from $\texttt{fish}^{(t)}$ according to mortality rate of fish μ_f.

8. “Summary statistics submodule”: Calculates various summary statistics over the human population and stores them in a array together with the environment variables described in Table B.

A.4 Design concepts

Emergent behavior: The model does not exhibit emergent behavior. The pivotal characteristic of heterogeneous worm burden in individuals is imposed by the distribution of individual worm acquiring parameters as described in further detail in Section A.5.1.

Stochasticity: Stochasticity is introduced by using pseudorandom numbers in every submodule as well as during initialization. Stochasticity does play an important role for stochastic extinction.

Observation: The state variables of the environment are stored for every time step over the simulation period. For the population of human individuals, various summary statistics are calculated and stored for time $t + dt$ after completing all the manipulations in time step t by the summary statistics submodule described in Section A.7.8. The entire population of individuals can be stored for all time steps as well but this option is usually turned off because of memory requirements.

The model does not make use of the further concepts described by ODD which are adaption, objectives, learning, prediction, sensing and interaction at the level of individuals as well as collectives.

A.5 Initialization

In the initialization phase, two 2-dimensional arrays are created. The first array, `humans`, contains an array for each human which tracks the variables listed in the upper part of Table B. The second array, `pop`, is initialized to contain as many vectors as there will be time steps throughout the model run. Each secondary array in `pop` stores the environment variables listed in the lower part of Table B as well as various

summary statistics that are calculated over the population of humans, for example the mean worm burden. This array is returned after a model run and used to produce the time series plots. The first element of this array will be populated with values as described in the following parts of this section.

A.5.1 Initialization of individuals in the humans array

The variable $\texttt{sex}_i$ is set randomly to 0 or 1, where the value 1 represents a male and is assigned with a probability given by parameter `proportion_male`. Given $\texttt{sex}_i$, each individual is assigned $\texttt{age}_i^{(0)}$ in days with probabilities given by vector parameters `population_distribution_male` and `population_distribution_female`. Individuals aged above `minimum_age_for_worm_infection` are assigned random worm counts $\texttt{worms}_i^{(0)}$ as specified by the vector parameters `initial_worm_distribution_probabilities` and `initial_worm_distribution_values`. Individuals aged above `minimum_age_for_worm_infection` have $\texttt{eating}_i^{(0)}$ set to 1. Furthermore, $\texttt{worms\_days}_i^{(0)}$ and $\texttt{MDA\_treatments}_i^{(0)}$ is set to 0 for all individuals, $\texttt{beta\_multiplier}_i^{(0)}$ is set to 1 for all individuals, and $\texttt{latrine}_i^{(0)}$ is set to 0 or 1 with probability `latrine_coverage`. We do currently not differentiate between latrine availability and latrine use and therefore set $\texttt{latrine}_i^{(0)}$ equal to $\texttt{latrine\_use}_i^{(0)}$.

The mechanism by which $\beta_{hf,i}$ is distributed among individuals is specified by the parameter `beta_hf_distribution`, with the options being `gamma` and `empirical_worms`. Because of the pivotal role of this distribution, we say that these options imply different *transmission models*. Each of the transmission models requires specific parameters:

- `gamma`: The $\beta_{hf,i}$ of individuals is drawn from a gamma distribution parameterized by `beta_hf_mean` and `beta_hf_variance`.
- `empirical_worms`: The $\beta_{hf,i}$ of individuals is drawn from a distribution determined by the vector parameters `initial_worm_distribution_probabilities` and `initial_worm_distribution_values`. First a worm count $\tilde{w}_i$ is drawn for each individual from the worm counts given by these two vectors. Subsequently, the individual's $\beta_{hf,i}$ is derived from the drawn $\tilde{w}_i$ using the relation
$$\log_{10}(\beta_{hf,i}) = \texttt{beta\_empirical\_worms\_a} + \texttt{beta\_empirical\_worms\_b} \times \log(\tilde{w}_i). \quad (1)$$

If the option `align_beta_hf_to_initial_worm_burden` is set to `True`, the $\beta_{hf,i}$ across individuals is distributed such that individuals with higher $worms_i^{(t)}$ are assigned a higher $\beta_{hf,i}$ with the intention of reaching equilibrium quicker.

A.5.2 Initialization of reservoir hosts

The number of worms in dogs and cats are initialized as

$$\texttt{dogs}^{(0)} = \texttt{N\_dogs} \times \texttt{initial\_worms\_per\_dog} \quad (2)$$

and

$$\texttt{cats}^{(0)} = \texttt{N\_cats} \times \texttt{initial\_worms\_per\_cat}, \quad (3)$$

where the resulting values are rounded to the closest integer. Similarly, the number of infected fish and infected snails are initialized as

$$\texttt{fish}^{(0)} = \texttt{N\_fish} \times \texttt{initial\_prevalence\_fish} \quad (4)$$

and

$$\texttt{snails}^{(0)} = \texttt{N\_snails} \times \texttt{initial\_prevalence\_snails}, \tag{5}$$

where the resulting values are again rounded to the closest integer.

A.6 Input data

Input data is passed to the model as model parameters, which are described in the following sections and listed in Tables D and E. The parameters controlling the transmission to humans and for interventions are described in the respective submodule subsections and are different from the input data specified here in that they are fitted to data as described in the main text.

Parameter	Value	Description	Source
N	$15,000$	Number of humans	–
proportion_male	0.5013	Proportion of males in humans	United Nations [3]
population_distribution_male	Vector of size 100×365	Proportion of males having the age in days corresponding to the vector index	United Nations [3]/interpolation
population_distribution_female	Vector of size 100×365	Proportion of females having the age in days corresponding to the vector index	United Nations [3]/interpolation
mortality_male	Vector of size 100×365	Mortality rate of males of the age in days corresponding to the vector index	United Nations [3]/interpolation
mortality_female	Vector of size 100×365	Mortality rate of females of the age in days corresponding to the vector index	United Nations [3]/interpolation
initial_worm_distribution_values	Vector of size 138	Worm counts which are assigned randomly initial population	2012 field data (contains 138 distinct EPG values)
initial_worm_distribution_probabilities	Vector of size 138	Probabilities with which worm counts at corresponding index in initial_worm_distribution_values are assigned	2012 field data (contains 138 distinct EPG values)
educ_decay	0.97	Base used in decay of education campaign effect in equation 10	2012 field data (contains 138 distinct EPG values)
max_worms_per_person	10^5	Maximum number of worms a human can harbor	Expert opinion
minimum_age_for_worm_infection	2×365 days	Minimum age at which humans eat potentially infectious fish	Expert opinion
epg_classification_bin_edges	$[0, 1, 10^3, 10^4, 10^6]$	Bin edges in EPG for classification of worm burden into low, moderate and high	WHO [4]
latrine_coverage	0.4496	Proportion of individuals with access to latrine at initialization	2012 field data
pregnancy_age_bins	Vector of size 8	Age bin edges measured in days for pregnancy proportions, see Table G	[5]
pregnancy_proportions	Vector of size 8	Pregnancy proportions in age groups specified by pregnancy_age_bins, see Table G	[5]

Table D. Complete list of input parameters.

Parameter	Value	Description	Source
N_dogs	2,500	Number of dogs, estimated to be one sixth of human population N	Expert opinion [6]
initial_worms_per_dog	0.53	Initial average number of worms per dog	2012 field data
N_cats	2,500	Number of cats, estimated to be one sixth of human population N	Expert opinion [6]
initial_worms_per_cat	5.63	Initial average number of worms per cat	2012 field data
N_snails	1,500,000	Population size of snails, estimated to be 100 times larger than the human population N	Expert opinion
initial_prevalence_snails	0.0029	Initial prevalence of parasite-positive snails	2012 field data
N_fish	150,000	Population size of snails, estimated to be 10 times larger than the human population N	Expert opinion
initial_prevalence_fish	0.2691	Initial prevalence of parasite-positive fish	2012 field data
feces_gram_per_day_human	100	Weight of feces a human excretes per day in gram	Expert opinion, [7]
feces_gram_per_day_dog	16.6	Weight of feces a dog excretes per day in gram	Expert opinion, [7]
feces_gram_per_day_cat	5	Weight of feces a cat excretes per day in gram	Expert opinion, [7]
education_minimum_age	0	Age in days above which an education campaign has an effect on safe fish consumption	Expert opinion
MDA_minimum_age	6×365	Age in days above which humans are included in MDA distribution campaigns	Expert opinion
μ_{ph}	$1/(10 \times 365)$	Mortality rate of parasites in humans per day	Previous modelling [8]
μ_{pd}	$1/(4 \times 365)$	Mortality rate of parasites in dogs per day	Previous modelling [8]
μ_{pc}	$1/(4 \times 365)$	Mortality rate of parasites in cats per day	Previous modelling [8]
μ_f	$1/(2.5 \times 365)$	Mortality rate of fish per day	Previous modelling [8]
μ_s	$1/(1 \times 365)$	Mortality rate of snails per day	Previous modelling [8]

Table E. Complete list of input parameters.

A.6.1 Human demography parameters

We set the population size of humans N to 15,000 corresponding to the population size at the site of data collection. We use 2015 data of the United Nations (UN) Demographic Statistics for Laos [3] to derive the values of human demography parameters proportion_male, population_distribution_male, population_distribution_female, mortality_male, and mortality_female. We choose to use national demographic data instead of the demographic characteristics from study participants due to a lack of detailed demographic data for the region. The UN data is processed in the same manner for males and females in which we apply slight modifications to achieve a monotonically decreasing size of age groups with increasing age. This involves evenly distributing the population up to an age of twenty years as well as a slight rearrangement of the population aged over 90 years under the assumption that individuals can attain an age of at most 100 years. This results in vectors population_distribution_male and population_distribution_female with 100×365 elements giving the proportion of the population having a specific age in days. From these vectors, we calculate the age-dependent daily probability of death required in order to keep the age distribution steady over model runs with

$$\texttt{mortality\_male}_i = 1 - \frac{\texttt{population\_distribution\_male}_{i+1}}{\texttt{population\_distribution\_male}_i} \tag{6}$$

for $i < (365 \times 100) - 1$ and $\texttt{mortality\_male}_{(365\times100)-1} = 1$. Age-dependent probabilities of death for females are obtained analogously. The age distribution and daily mortality probability for females is depicted in Fig F.

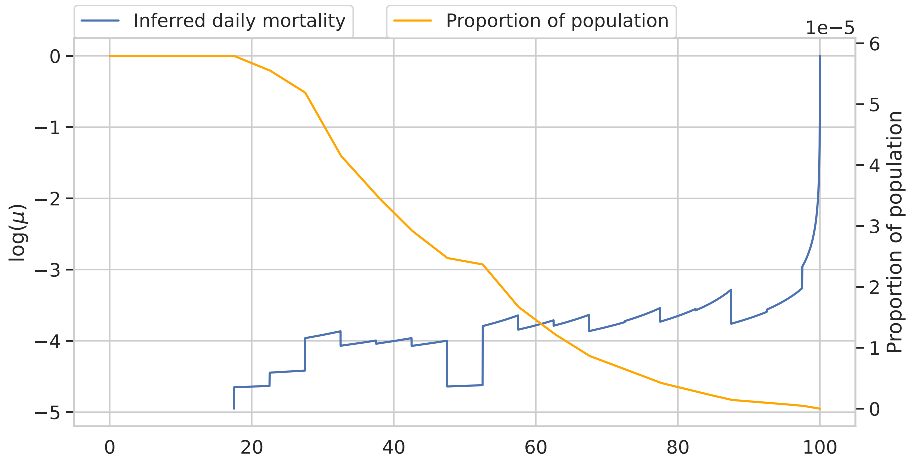

Fig F. Age distribution of females in modified 2015 UN data and inferred mortality rates.

The frequency of pregnancy, which leads to exclusions from MDA campaigns, is provided by the parameters pregnancy_age_bins and pregnancy_proportions, which contain the values listed in Table G.

A.6.2 Transformation worms to eggs per gram

We use a power law derived from multiple purging and autopsy studies to transform EPG to worm counts in individuals and vice versa [9]:

Age range	Proportion pregnant
0–14	0
15–19	0.0825
20–24	0.165
25–29	0.18
30–34	0.1425
35–39	0.0825
40–44	0.03
45+	0

Table G. Proportion of females pregnant at any given point in time per age group derived from fertility rates from 2005 [5].

$$\begin{aligned} EPG &= \rho(\text{worms}) \\ &= 20 \times \text{worms}^{-0.82} \end{aligned} \tag{7}$$

In absence of data for animals, we assume that a parasite produces the same number of EPG in cats and dogs.

A.6.3 Worm distribution in humans and feces quantities

The number of worms present in the initial population parameterized by `initial_worm_distribution_probabilities` and `initial_worm_distribution_values` are obtained by transforming the EPG data collected in the 2012 survey using the inverse ρ^{-1} of the function given in equation (7). We set the maximum number of worms a human can harbor `max_worms_per_person` to 10^5 and the `minimum_age_for_worm_infection` to 2×365 days assuming younger children do not consume potentially contaminated dishes. We use WHO recommendations to categorize the intensity of *O. viverrini* infection as light ($1 - 999$ EPG), moderate ($1,000 - 9,999$ EPG) or heavy ($\geq 10,000$ EPG) [10]. This classification scheme is passed to the model in the form of the vector parameter `epg_classification_bin_edges` containing binning edges. We set `feces_gram_per_day_human` to 100 grams. Based on the estimation that a dog weighs one sixth of the human and stool production is proportional to body weight, we set `feces_gram_per_day_dog`to 16.6 grams. Assuming cats have 5% of human body weight, we set `feces_gram_per_day_cat` to 5 grams.

A.6.4 Further parameters

We set `latrine_coverage` to 0.4406, which equals the coverage over the entire study population in 2012.

A.6.5 Reservoir host population parameters and worm mortality

We parameterize the number of dogs `N_dogs` and the number of cats `N_cats` to be one sixth of the human population size `N` based on questioning individuals in the study population [6]. We calculate `initial_worms_per_dog` and `initial_worms_per_cat` from EPG data collected for cats and dogs at the study site in 2012. Transforming the EPG count from individual animals using ρ^{-1} and taking the average results in 0.53 `initial_worms_per_dog` and 5.63 `initial_worms_per_cat`.

We set the population size of snails `N_snails` to be 100 times the population size of humans `N` and the population size of fish `N_fish` to be 10 times that of humans `N`.

These numbers are not based on data but on rough guesses and likely far from the true values. However, we expect misestimates of these numbers not to have an effect on the system's dynamics when the transmission parameter values are fitted to data. We set the `initial_prevalence_snails` to 0.0029 and `initial_prevalence_fish` to 0.2691 based on samples of fish and snails taken at the study site in 2012.

We set the worm mortality μ_{ph} in humans based on a life expectancy of the parasite of 10 years to $\frac{1}{10\times365}$. For other hosts, the life expectancy of the host as opposed to the life expectancy of the parasite is the limiting factor. We set the mortality parameters analogously assuming life expectancies of 4 years for cats and dogs, 2.5 years for fish and 1 year for snails [8].

A.7 Submodules

A.7.1 MDA submodule

The MDA submodule checks if an MDA round is scheduled within the time interval $[t, t + dt)$ with the timings of MDA campaigns specified by the vector parameter `MDA_timing`. Only one round of MDA can be conducted in a time step.

If an MDA round is scheduled in the current time step, individuals eligible for MDA treatment are determined. These are all individuals aged above `MDA_minimum_age` who are not pregnant. Pregnancy is randomly assigned in each MDA round to female individuals according to parameters `pregnancy_age_bins` and `pregnancy_prortions`, which specify the proportion of pregnant women for different age groups. This randomly assigned pregnancy is only a temporary variable used to determine MDA eligibility and is not stored at the individual level. Therefore the model is not well-suited for modelling MDA campaigns taking place more frequently than once a year. The MDA coverage levels specified through `MDA_coverage` apply to the population of individuals eligible for MDA.

Subsequently, it is determined which eligible individuals receive MDA depending on the parameter `MDA_strategy`, for which there are two choices:

- "`random`" assigns MDA randomly to individuals with the probability of the coverage specified for the current MDA round in the vector parameter `MDA_coverage`, which contains the coverage of each MDA round listed in `MDA_timing`.

- "`gaussian_coppula`" assigns individuals a probability $p_{m,i}$ of adhering to an MDA campaign. The probabilities are determined at the moment when the first MDA campaign is carried out and stored in the vector `MDA_adherence_probabilities`. Individuals keep their $p_{m,i}$ campaign over all following MDA campaigns. $p_{m,i}$ is drawn from a beta distribution with a mean specified by the parameter `MDA_coverage` and variance specified by the parameter `MDA_variance`. Correlation between $p_{m,i}$ and the individual transmission parameters, $\beta_{hf,i}$, is introduced through a Gaussian copula with the following steps:
  - The $\beta_{hf,i}$ are mapped to the interval [0,1] using the cumulative distribution function of the gamma distribution that generated the $\beta_{hf,i}$, resulting in a vector of values X_U.
  - X_U is then mapped to X_N with the standard normal quantile function.
  - A new set of normally distributed random values Y_N is generated which are correlated with X_N using the function

$$Y_N = X_N * \rho + \varepsilon \times \sqrt{1 - \rho^2}, \tag{8}$$

where ρ is the correlation coefficient between the latent Gaussian variables in the copula and is specified through the parameter `MDA_copula_correlation` and ε are random draws from a standard normal distribution.

  – Y_N is mapped to Y_U on the interval [0,1] using the standard normal cumulative density function.
  – The $p_{m,i}$ are generated by transforming Y_U using the quantile function of the beta distribution with mean and variance specified by parameters `MDA_coverage` and `MDA_variance`.

In each round of MDA, individuals receive MDA with probability $p_{m,i}$, provided they are eligible for MDA as described above.

This option currently only works with the gamma-distributed $\beta_{hf,i}$ but could also be implemented with the empirical model.

For individuals receiving MDA, $\texttt{worms}_i^{(t)}$ is set to 0 with immediate effect on transmission and $\texttt{MDA\_treatment}_i^{(t)}$ is increased by 1.

A.7.2 Education submodule

Checks if an education round is scheduled within the time interval $[t, t + dt)$ with education timings specified in days by vector parameter `education_timing`. Only one round of education can be conducted in a time step.

If an education round is scheduled in the current time step, individuals eligible for education are determined. These are all individuals aged above `education_minimum_age`. There are two ways in which individuals that eventually receive education are determined depending on the parameter `education_strategy`:

- "`random`" selects individuals randomly with the probability of the coverage specified for the current education round in the vector parameter `education_coverage`, which contains the coverage of each education round listed in `education_timing`. For individuals receiving education, $\texttt{beta\_multiplier}_i^{(t)}$ is multiplied with the education efficacy of the current education round specified in the vector parameter `educ_efficacy` with an immediate effect on transmission.

- "`gaussian_coppula`" assigns individuals an education factor e_i that determines the effect an education campaign has on them. The e_i are determined when the first education campaign is carried out and are stored in the vector `edcucation_adherences`. The factors then stay fixed over time for individuals. They are drawn from a beta distribution with a mean specified by the parameter `education_efficacy_mean` and variance specified by the parameter `education_efficacy_variance`. Correlation between e_i and the individual transmission parameters, $\beta_{hf,i}$, is introduced through a Gaussian copula with the following steps:

  – The $\beta_{hf,i}$ are mapped to the interval [0,1] using the cumulative distribution function of the gamma distribution that generated the $\beta_{hf,i}$, resulting in a vector of values X_U.
  – X_U is then mapped to X_N with the standard normal quantile function.
  – A new set of random values Y_N is generated which are correlated with X_N using the function
$$Y_N = X_N * \rho + \varepsilon \times \sqrt{1 - \rho^2}, \quad (9)$$

where ρ is the correlation coefficient between the latent Gaussian variables in the copula and is specified through the parameter edcuation_copula_correlation and ε are random draws from a standard normal distribution.

– Y_N is mapped to Y_U on the unit interval [0,1] using the standard normal cumulative density function.
– The e_i are generated by transforming Y_U using the quantile function of the beta distribution with mean and variance specified by parameters education_efficacy_mean and education_efficacy_variance.

In each round of education, individuals eligible for education have their $\texttt{beta\_multiplier}_i^{(t)}$ multiplied with their individual education factor e_i with an immediate effect on transmission.

When considering waning effects of the education campaign, we model a waning effect of the education campaign factor by updating each individual's $\texttt{beta\_multiplier}_i^{(t)}$ at each time step using the formula

$$\texttt{beta\_multiplier}_i^{(t+1)} = 1 - \texttt{educ\_decay}^{\frac{1}{\texttt{timesteps\_per\_year}}} \times \left(1 - \texttt{beta\_multiplier}_i^{(t)}\right), \quad (10)$$

which results in an exponential-like decay of the effect of the education campaigns. We choose an arbitrary value of 0.97 for educ_decay for the analysis in the main paper (in absence of any data on this). The results of the analysis are not changed if a different value for educ_decay is chosen as long as it is greater than 0.9.

A.7.3 Latrines submodule

Checks if a change in latrine coverage is scheduled within the time interval $[t, t + dt)$ with latrine change timings specified in days by vector parameter latrine_change_timing. If this is the case, latrine coverage is changed according the scheme specified by the parameter latrine_change_plan and latrine_change_coverage. There are two choices latrine_change_plan: random assigns latrines randomly among all individuals in the population with coverage latrine_change_coverage. This means some individuals can lose access to latrines; addition calculates the difference in coverage between latrine_change_coverage and the current coverage, and randomly distributes latrines within the population that currently has no latrines such that the population has the latrine coverage specified by latrine_change_coverage after the intervention. Removing latrines with the option addition is currently not supported.

A.7.4 Human worms submodule

Checks which individuals reach the minimum_age_for_worm_infection within the time interval $[t, t + dt)$ and randomly sets their $\texttt{eating}_i^{(t)}$ state variable to 0 or 1 with an immediate effect on transmission.

Calculates the number of worms

$$\texttt{worms}_i^{(t+dt)} = \min\left(\texttt{worms}_i^{(t)} - \mathcal{M}_{ph,i}^{(t)} + \mathcal{E}_{ph,i}^{(t)}, \texttt{max\_worms\_per\_human}\right) \quad (11)$$

for each individual at time $t + dt$ based on the values of state variables at time t. Parasite mortality within humans occurs by subtracting

$$\mathcal{M}_{ph,i}^{(t)} \sim \text{Binomial}\left(\texttt{worms}_i^{(t)}, 1 - e^{-dt \times \mu_{ph}}\right). \quad (12)$$

The acquisition of new worms occurs by adding

$$\mathcal{E}_{ph,i}^{(t)} \sim \text{Poisson}\left(dt \times \beta_{hf,i} \times \texttt{beta\_multiplier}_i^{(t)} \times \frac{\texttt{fish}}{\texttt{N\_fish}}\right), \tag{13}$$

for individuals with $\texttt{eating}_i^{(t)} = \texttt{True}$, whereas for individuals with $\texttt{eating}_i^{(t)} = \texttt{False}$, $\mathcal{E}_{ph,i}^{(t)}$ equals 0. Additionally, this module integrates the mean number of worms over time in order to track the `worm_days` of individuals:

$$\texttt{worms\_days}_i^{(t+dt)} = \texttt{worms\_days}_i^{(t)} + dt \times \frac{\texttt{worms}_i^{(t)} + \texttt{worms}_i^{(t+dt)}}{2}. \tag{14}$$

Finally, the number of EPG in each individual's stool is calculated using the transformation function described in Section A.6.2,

$$\texttt{epg}_i^{(t+dt)} = \rho\left(\texttt{worms}_i^{(t+dt)}\right), \tag{15}$$

where the output of the transformation function $\rho(\cdot)$ is rounded to the closest integer.

A.7.5 Demography submodule

The probability of death in the current time step for each individual is read from the vector parameters `mortality_male` and `mortality_female` depending on $\texttt{age}_i^{(t)}$ and $\texttt{sex}_i$. If the time step dt is greater than one, the mortality rates which are initially supplied as daily values, get converted to mortality rates per time step. A Bernoulli random variable based on the individuals' probability is drawn determining whether they die in the current time step. In an intermittent step, dt is added to each individual's age $\texttt{age}_i^{(t)}$. All individuals dying in the current time step are replaced by newborn individuals by resetting state variables $\texttt{age}_i^{(t+dt)}$ to a random number between 0 and dt with a uniform distribution, $\texttt{worms}_i^{(t)}$, $\texttt{worm\_days}_i^{(t)}$, and $\texttt{MDA\_treatments}_i^{(t)}$ to 0, $\texttt{eating}_i^{(t)}$ to `False`, and $\texttt{beta\_multiplier}_i^{(t)}$ to 1.

A.7.6 Reservoir hosts submodule

The total number of worms in dogs updated according to

$$\texttt{dogs}^{(t+dt)} = \texttt{dogs}^{(t)} - \mathcal{M}_{pd}^{(t)} + \mathcal{E}_{pd}^{(t)}, \tag{16}$$

where the mortality of worms in dogs is given by

$$\mathcal{M}_{pd}^{(t)} \sim \text{Binomial}\left(\texttt{dogs}^{(t)}, 1 - e^{-dt \times \mu_{pd}}\right), \tag{17}$$

and acquisition of new worms in dogs is given by

$$\mathcal{E}_{pd}^{(t)} \sim \text{Poisson}\left(dt \times \beta_{df} \times \texttt{N\_dogs} \times \frac{\texttt{fish}}{\texttt{N\_fish}}\right). \tag{18}$$

Analogously, the number of worms in cats is updated according to

$$\texttt{cats}^{(t+dt)} = \texttt{cats}^{(t)} - \mathcal{M}_{pc}^{(t)} + \mathcal{E}_{pc}^{(t)}, \tag{19}$$

where the mortality of worms in cats is given by

$$\mathcal{M}_{pc}^{(t)} \sim \text{Binomial}\left(\texttt{cats}^{(t)}, 1 - e^{-dt \times \mu_{pc}}\right), \tag{20}$$

and acquisition of new worms in cats is given by

$$\mathcal{E}_{pc}^{(t)} \sim \text{Poisson}\left(dt \times \beta_{cf} \times \texttt{N\_cats} \times \frac{\texttt{fish}}{\texttt{N\_fish}}\right). \tag{21}$$

A.7.7 Intermediate hosts submodule

First, the sum of total eggs produced by humans, dogs and cats is calculated:

$$\begin{aligned}
\Xi_h^{(t)} &= \sum_{i=1}^{\mathtt{N}} \rho\left(\mathtt{worms}_i^{(t)}\right) \times \left(1 - \mathtt{latrine_use}_i^{(t)}\right) \times \mathtt{feces_gram_per_day_human}, \\
\Xi_d^{(t)} &= \rho\left(\mathtt{dogs}^{(t)}/\mathtt{N_dogs}\right) \times \mathtt{N_dogs} \times \mathtt{feces_gram_per_day_dog}, \\
\Xi_c^{(t)} &= \rho\left(\mathtt{cats}^{(t)}/\mathtt{N_cats}\right) \times \mathtt{N_cats} \times \mathtt{feces_gram_per_day_cat}.
\end{aligned} \tag{22}$$

The number of infected snails is now updated with

$$\mathtt{snails}^{(t+dt)} = \min\left(\mathtt{snails}^{(t)} - \mathcal{M}_s^{(t)} + \mathcal{E}_s^{(t)}, \mathtt{N\_snails}\right), \tag{23}$$

where the mortality of infected snails is given by

$$\mathcal{M}_s^{(t)} \sim \text{Binomial}\left(\mathtt{snails}^{(t)}, 1 - e^{-dt \times \mu_s}\right), \tag{24}$$

and the emergence of newly infected snails is given by

$$\mathcal{E}_s^{(t)} \sim \text{Poisson}\left(dt \times \left(\frac{\beta_{sh}\Xi_h^{(t)}}{\mathtt{N}} + \frac{\beta_{sd}\Xi_d^{(t)}}{\mathtt{N\_dogs}} + \frac{\beta_{sc}\Xi_c^{(t)}}{\mathtt{N\_cats}}\right) \times \left(\mathtt{N\_snails} - \mathtt{snails}^{(t)}\right)\right). \tag{25}$$

Finally, the number of infected fish is determined by

$$\mathtt{fish}^{(t+dt)} = \min\left(\mathtt{fish}^{(t)} - \mathcal{M}_f^{(t)} + \mathcal{E}_f^{(t)}, \mathtt{N\_fish}\right), \tag{26}$$

where the mortality of infected fish is given by

$$\mathcal{M}_f^{(t)} \sim \text{Binomial}\left(\mathtt{fish}^{(t)}, 1 - e^{-dt \times \mu_f}\right), \tag{27}$$

and the emergence of newly infected fish is given by

$$\mathcal{E}_s^{(t)} \sim \text{Poisson}\left(dt \times \beta_{fs} \times \frac{\mathtt{snails}^{(t)}}{\mathtt{N\_snails}} \times \left(\mathtt{N\_fish} - \mathtt{fish}^{(t)}\right)\right). \tag{28}$$

A.7.8 Summary statistics submodule

Stores summary statistics for the population of humans. This includes the mean age of humans, the latrine coverage and usage, the number of people consuming raw or undercooked fish, the number of people above `minimum_age_for_worm_infection`, the total number of worms in humans, the number of humans currently infected, the maximum number of worms any human carries in the population and the variance of number of worms humans carry. For EPG in individuals, the mean, the variance and maximum values are stored. Additionally, the number of individuals having light, moderate or high worm burden as specified by the parameter `epg_classification_bin_edges` and the mean EPG among individuals within each of those groups are stored.

B Population-based model description

The population-based model (PBM) is defined by the following system of differential equations:

$$\frac{\mathrm{d}w_h}{\mathrm{d}t} = \beta_{hf} i_f - \mu_{ph} w_h, \tag{29a}$$

$$\frac{\mathrm{d}w_d}{\mathrm{d}t} = \beta_{df} i_f - \mu_{pd} w_d, \tag{29b}$$

$$\frac{\mathrm{d}w_c}{\mathrm{d}t} = \beta_{cf} i_f - \mu_{pc} w_c, \tag{29c}$$

$$\frac{\mathrm{d}i_s}{\mathrm{d}t} = (\beta_{sh} w_h + \beta_{sd} w_d + \beta_{sc} w_c)(1 - i_s) - \mu_s i_s, \tag{29d}$$

$$\frac{\mathrm{d}i_f}{\mathrm{d}t} = \beta_{fs} i_s (1 - i_f) - \mu_f i_f. \tag{29e}$$

The parameters and the values used for the analyses are listed in Table H. Whenever applicable, we use the same values as in the agent-based models. Further information on the model can be found in the original publications [8, 11].

Parameter	Description	Dimension	Value
N_h	Population size of humans	Animals	$15,000$
N_d	Population size of dogs	Animals	$2,500$
N_c	Population size of cats	Animals	$2,500$
N_s	Population size of snails	Animals	$1,500,000$
N_f	Population size of fish	Animals	$150,000$
μ_{ph}	Per capita death rate of adult parasites in humans (includes additional mortality due to death of humans)	$\frac{1}{\text{Time}}$	$\frac{1}{10\times365}$
μ_{pd}	Per capita death rate of adult parasites in dogs (includes additional mortality due to death of dogs)	$\frac{1}{\text{Time}}$	$\frac{1}{4\times365}$
μ_{pc}	Per capita death rate of adult parasites in cats (includes additional mortality due to death of cats)	$\frac{1}{\text{Time}}$	$\frac{1}{4\times365}$
μ_s	Per capita death rate of snails	$\frac{1}{\text{Time}}$	$\frac{1}{1\times365}$
μ_f	Per capita death rate of fish including mortality through fishing by humans	$\frac{1}{\text{Time}}$	$\frac{1}{2.5\times365}$
β_{hf}	Transmission rate from infectious fish to humans per person per fish	$\frac{1}{\text{Time}\times\text{Animals}}$	0.394
β_{df}	Transmission rate from infectious fish to dogs per dog per fish	$\frac{1}{\text{Time}\times\text{Animals}}$	0.003
β_{cf}	Transmission rate from infectious fish to cats per cat per fish	$\frac{1}{\text{Time}\times\text{Animals}}$	0.187
β_{sd}	Infection rate of snails per parasite in a dog host	$\frac{1}{\text{Time}\times\text{Animals}}$	1.721×10^{-8}
β_{sc}	Infection rate of snails per parasite in a cat host	$\frac{1}{\text{Time}\times\text{Animals}}$	1.721×10^{-8}
β_{sh}	Infection rate of snails per parasite in a human host	$\frac{1}{\text{Time}\times\text{Animals}}$	1.721×10^{-8}
β_{fs}	Infection rate of fish per infected snail	$\frac{1}{\text{Time}\times\text{Animals}}$	0.141

Table H. Parameters of the PBM.

C Additional figures

This section contains two plots with additional variables for the model runs with non-systematic and systematic adherence shown in the main text.

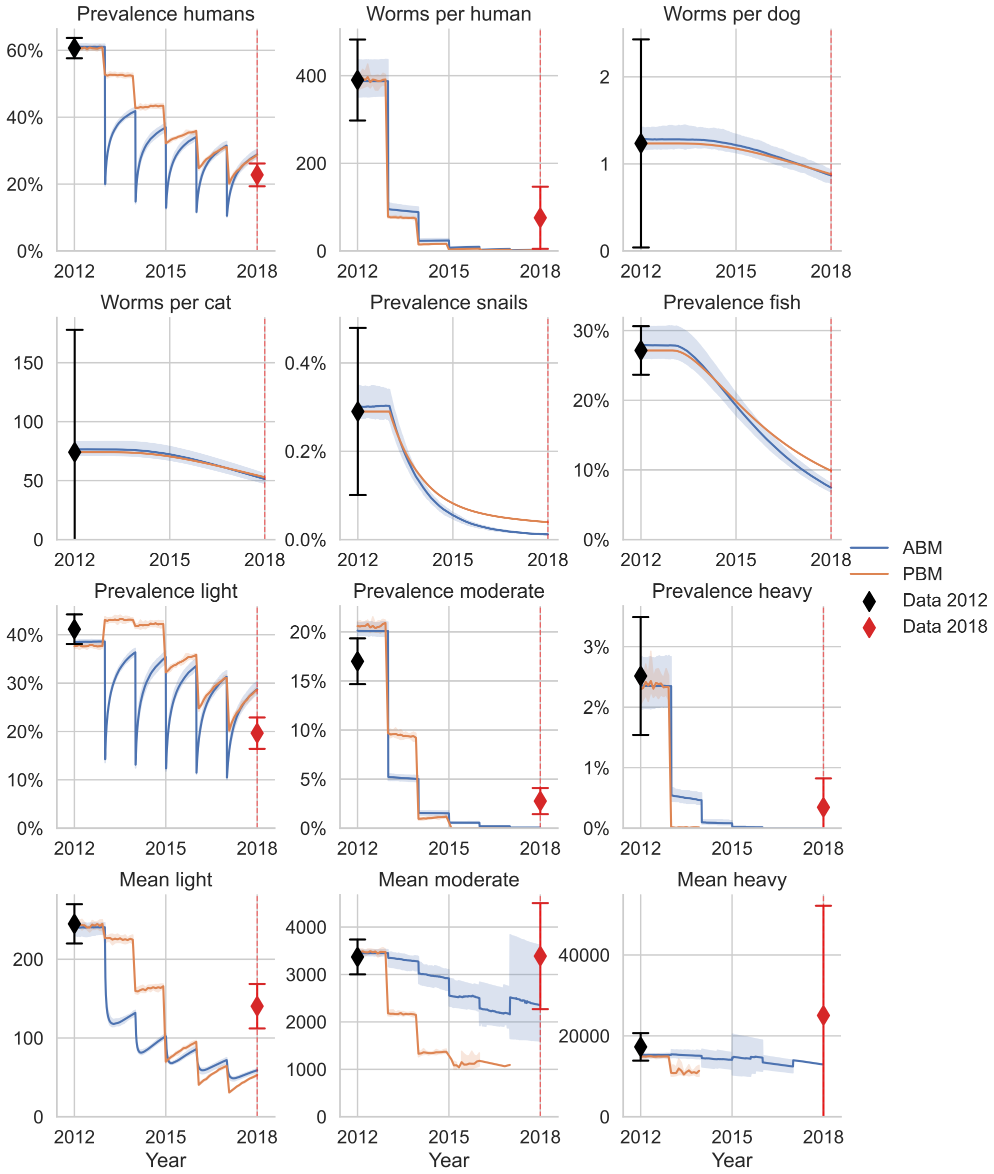

Fig I. Time series for various summary statistics as predicted by the models starting in equilibrium followed by five years of interventions (MDA, education campaign, improved sanitation, all without systematic adherence). Both models were calibrated to fit the 2012 data in equilibrium but not the 2018 data.

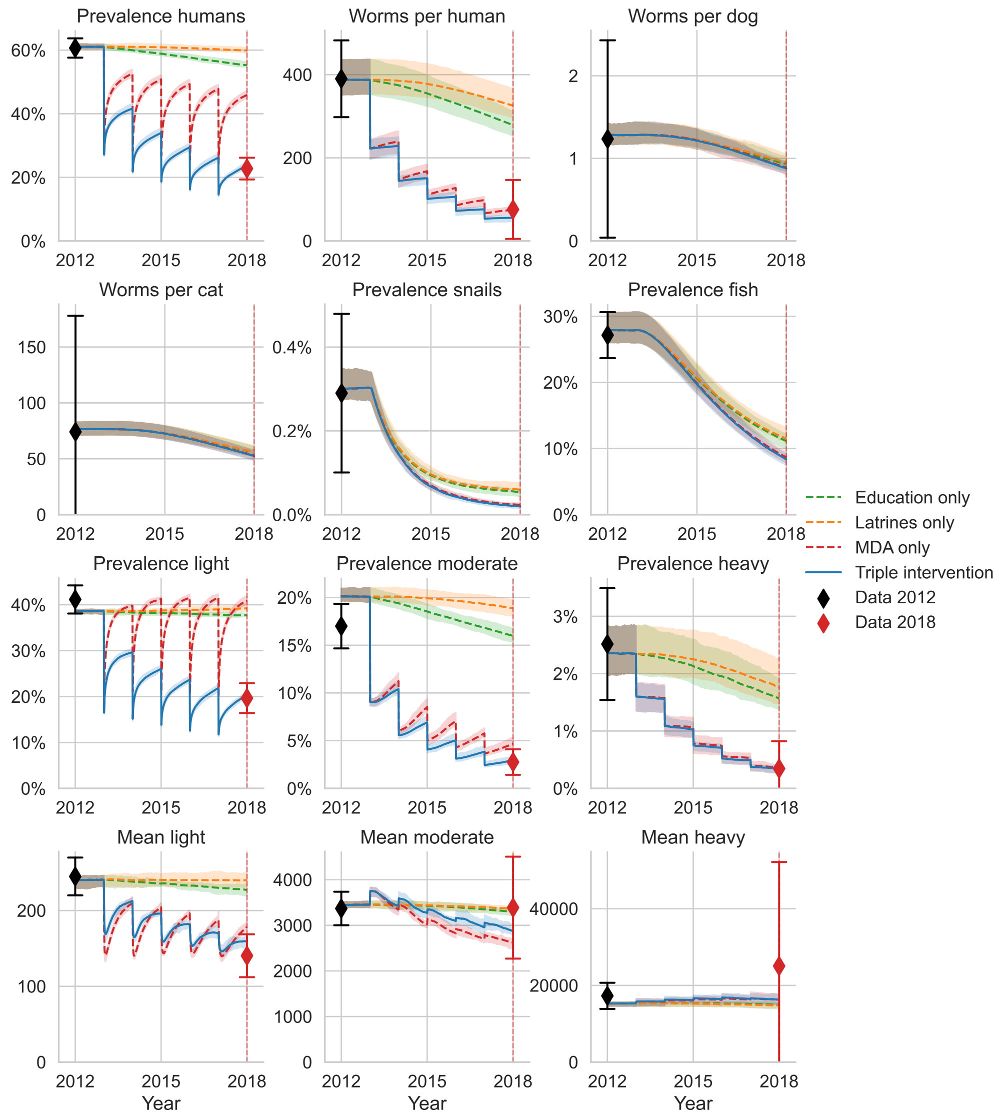

Fig J. Time series for various summary statistics as predicted by the ABM with fitted systematic adherence where all three interventions are implemented together or in isolation.

D Model variant with only humans as definitive hosts: Additional figures

This section contains the last two plot of the main text for the model runs without animals as definitive hosts. Animals were removed from the system by setting the transmission parameter from fish to non-human animals to zero and refitting the other transmission parameters. All the qualitative conclusions from the main text are not affected by this removal, although the exact times until the goals are achieved are slightly reduced.

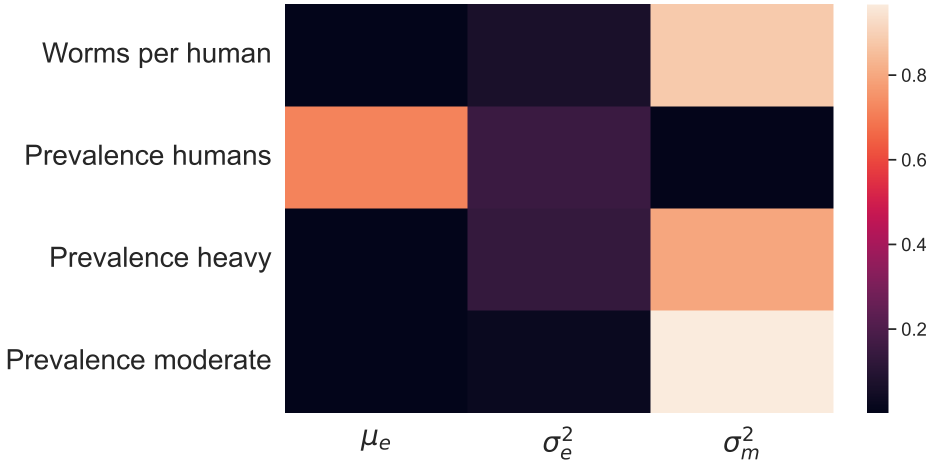

Fig K. First-order effects measured in the global sensitivity analysis of fitted intervention parameters (x-axis) on goodness of fit of the model to the objective summary statistics in the 2018 data for the model without non-human animals. The parameter controlling the mean effect of the education campaign, μ_e, has mostly a strong impact on the prevalence in humans. Systematic adherence to the education campaign, controlled through the parameter σ_e^2, affects prevalence, prevalence of heavy worm burden, and mean worm burden, though for each one of these outcomes, one of the other two studied parameters is of greater importance. Systematic adherence to the MDA campaign, controlled through the parameter σ_m^2, has a strong effect on the prevalence of moderate and heavy worm worm burden and consequently on the mean worm burden in the population.

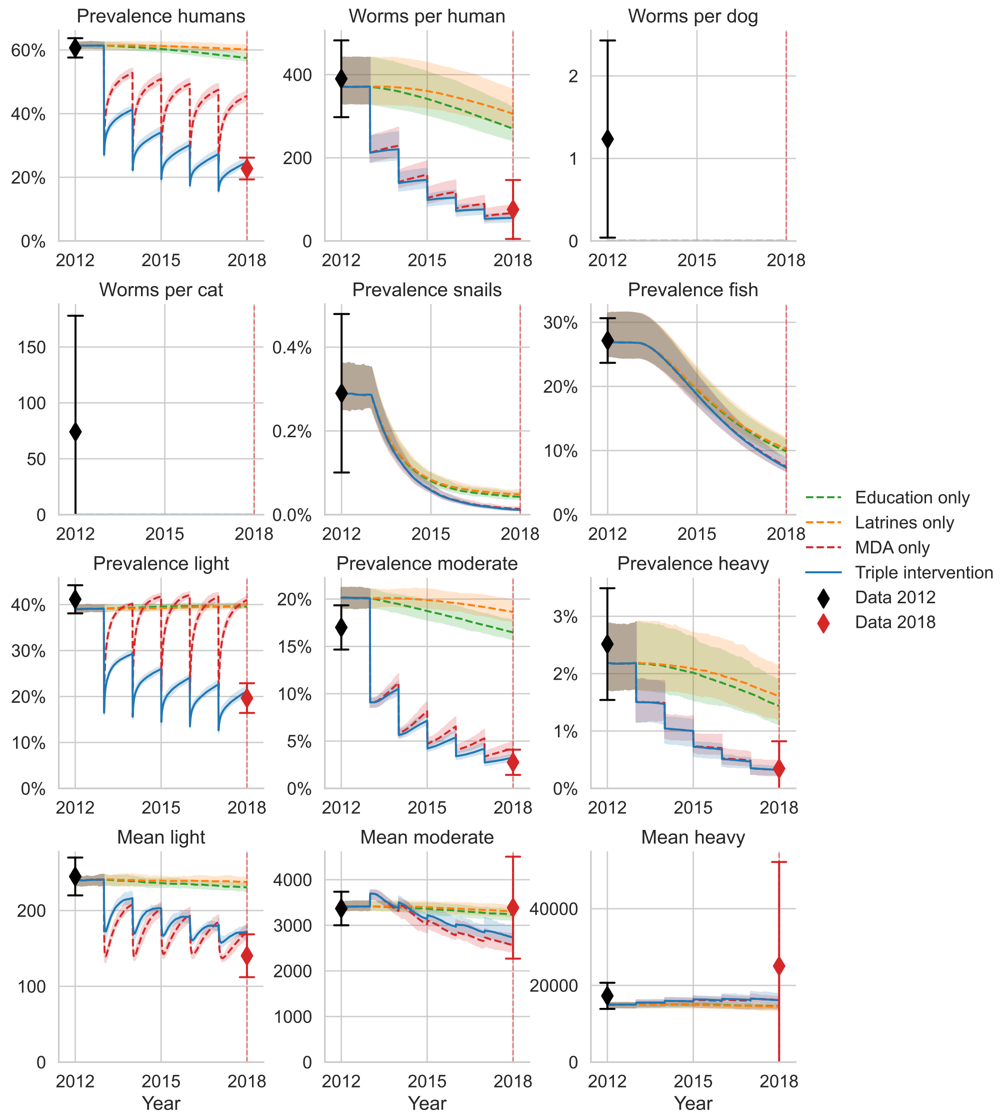

Fig L. Time series for various summary statistics as predicted by the ABM without non-human animals as definitive hosts with fitted systematic adherence where all three interventions are implemented together or in isolation.

References

1. Grimm V, Berger U, Bastiansen F, Eliassen S, Ginot V, Giske J, et al. A standard protocol for describing individual-based and agent-based models. Ecological modelling. 2006;198(1-2):115–126.

2. Grimm V, Railsback SF, Vincenot CE, Berger U, Gallagher C, DeAngelis DL, et al. The ODD protocol for describing agent-based and other simulation models: A second update to improve clarity, replication, and structural realism. Journal of Artificial Societies and Social Simulation. 2020;23(2).

3. United Nations Demographic Statistics Database. Population by age, sex and urban rural residence; 2022. Available from: http://data.un.org/Data.aspx?d=POP&f=tableCode%3A22 [cited 2022-10-14].

4. WHO Study Group on the Control of Foodborne Trematode Infections. Control of foodborne trematode infections. Report of a WHO study group. World Health Organization; 1995.

5. United Nations Demographic Statistics Database. Age-specific fertility rates, Total fertility and Mean age at childbearing; 2022. Available from: http://data.un.org/documentdata.aspx?id=319 [cited 2023-02-15].

6. Yerbi VL. Diversity of Helminth Infections in Humans and Animals of Rural Lao PDR [masters Thesis]. University of Basel, Switzerland; 2019.

7. Cummings JH, Bingham SA, Heaton KW, Eastwood MA. Fecal weight, colon cancer risk, and dietary intake of nonstarch polysaccharides (dietary fiber). Gastroenterology. 1992;103(6):1783–1789.

8. Bürli C, Harbrecht H, Odermatt P, Sayasone S, Chitnis N. Mathematical analysis of the transmission dynamics of the liver fluke, *Opisthorchis viverrini*. Journal of Theoretical Biology. 2018;439:181–194.

9. Crellen T, Haswell M, Sithithaworn P, Sayasone S, Odermatt P, Lamberton PH, et al. Diagnosis of helminths depends on worm fecundity and the distribution of parasites within hosts. Proceedings of the Royal Society B. 2023;290(1991):20222204.

10. Vonghachack Y, Odermatt P, Taisayyavong K, Phounsavath S, Akkhavong K, Sayasone S. Transmission of *Opisthorchis viverrini*, Schistosoma mekongi and soil-transmitted helminthes on the Mekong Islands, Southern Lao PDR. Infectious Diseases of Poverty. 2017;6(1):131.

11. Bürli C, Harbrecht H, Odermatt P, Sayasone S, Chitnis N. Analysis of interventions against the liver fluke, *Opisthorchis viverrini*. Mathematical Biosciences. 2018;303:115–125.
